# Oxidative Stress Biomarkers in Oral Mucosal Wound Healing and Photobiomodulation: Biochemical Pathways, Experimental Models, and Translational Perspectives

**DOI:** 10.3390/ijms27135763

**Published:** 2026-06-26

**Authors:** Ilija M. Dragojević, Bojana Kisić, Dijana Mirić, Aleksandra Ilić, Jelena T. Todić, Milena Kostić, Zlatibor Anđelković, Ljiljana Popović, Ljiljana Šubarić, Aleksandar Šubarić, Nadica S. Đorđević

**Affiliations:** 1 Institute of Biochemistry, Faculty of Medicine, University of Priština in Kosovska Mitrovica, 38220 Kosovska Mitrovica, Serbia; bojanabk2002@yahoo.com (B.K.); miric.dijana@gmail.com (D.M.); 2 Department of Preventive Medicine, Faculty of Medicine, University of Priština in Kosovska Mitrovica, 38220 Kosovska Mitrovica, Serbia; aleksandra.ilic@med.pr.ac.rs; 3 Department of Dentistry, Faculty of Medicine, University of Priština in Kosovska Mitrovica, 38220 Kosovska Mitrovica, Serbia; jelena.todic@med.pr.ac.rs (J.T.T.); emajel@gmail.com (L.Š.); nadica.djordjevic@med.pr.ac.rs (N.S.Đ.); 4 Clinic for Dental Medicine, Faculty of Medicine, University of Niš, 18000 Niš, Serbia; milena.kostic@medfak.ni.ac.rs; 5 Institute of Histology, Faculty of Medicine, University of Priština in Kosovska Mitrovica, 38220 Kosovska Mitrovica, Serbia; zlatibor.andjelkovic@med.pr.ac.rs; 6 Institute of Pathophysiology, Faculty of Medicine, University of Priština in Kosovska Mitrovica, 38220 Kosovska Mitrovica, Serbia; ljliljana.popovic@med.pr.ac.rs; 7 Faculty of Medicine, University of Priština in Kosovska Mitrovica, 38220 Kosovska Mitrovica, Serbia; subaricaleksandar@gmail.com

**Keywords:** photobiomodulation, low-level laser therapy, oral mucosal wound healing, oxidative stress, redox signaling, reactive oxygen species, oxidative stress biomarkers, redox-sensitive pathways

## Abstract

Oral mucosal repair is a redox-regulated process that may be impaired by diabetes, chronic inflammation, infection, and chemotherapy- or radiotherapy-induced oral mucositis. Reactive oxygen species (ROS) support host defense, epithelial migration, angiogenesis, extracellular matrix remodeling, and adaptive repair when their production is transient and compartmentalized. In contrast, persistent ROS promote lipid, protein, and DNA oxidation, mitochondrial dysfunction, and extracellular matrix damage. Photobiomodulation (PBM) is increasingly used to support oral tissue repair, but its effects should be interpreted as dose- and context-dependent redox modulation rather than as simple antioxidant activity. This narrative review synthesizes oxidative stress biomarkers and redox-sensitive pathways relevant to oral mucosal repair and PBM, including oxidant–antioxidant balance, lipid and protein oxidation, oxidative DNA damage, antioxidant defense, thiol/disulfide homeostasis, mitochondrial and NADPH oxidase-derived ROS, Nrf2/HO-1, NF-κB, HIF-1α/VEGF, MAPK/ERK, PI3K/Akt, and MMP/TIMP signaling. The review emphasizes the distinction between transient mitochondrial ROS/nitric oxide signaling and sustained NADPH oxidase-driven oxi-inflammatory stress. It proposes a practical redox-guided framework for biomarker selection, PBM response interpretation, and future study design, while noting that this framework remains conceptual and is not yet a validated clinical decision algorithm.

## 1. Introduction

Oral mucosal wounds are common clinical problems and useful biological models because they develop in a constantly changing environment shaped by saliva, microorganisms, mastication-related mechanical stress, diet, and immune surveillance. Although human oral mucosa is molecularly primed for rapid repair and often heals faster than skin, this advantage can be weakened by oxidative-stress-related conditions, including diabetes mellitus, chronic inflammation, infection, chemotherapy- or radiotherapy-induced oral mucositis, and systemic metabolic disease [1,2,3,4,5,6]. In these settings, successful healing requires more than epithelial closure; it also depends on inflammatory resolution, vascular normalization, appropriate extracellular matrix remodeling, microbial control, and restoration of mucosal barrier function [1,2,5].

Reactive oxygen species (ROS) are not merely by-products of tissue injury. In oral wound repair, their biological meaning depends on concentration, timing, cellular source, and anatomical compartment. Short-lived and locally restricted ROS signals support host defense, epithelial migration, angiogenic signaling, inflammatory-cell coordination, and adaptive stress responses. By contrast, sustained oxidant excess promotes lipid, protein, nucleic acid, mitochondrial, and extracellular matrix damage, thereby prolonging inflammation and impairing tissue restoration [7,8,9,10,11,12].

This distinction between physiological redox signaling and pathological oxidative stress is central to oral wound-healing research. Redox biomarkers are most informative when they are grouped by biological meaning rather than interpreted as isolated numerical changes. Global oxidant–antioxidant balance can be assessed using total oxidant status (TOS), total antioxidant capacity (TAC), and oxidative stress index (OSI); lipid oxidation by malondialdehyde (MDA) and 4-hydroxynonenal (4-HNE); protein oxidation by advanced oxidation protein products (AOPP) and protein carbonyls; DNA oxidation by 8-hydroxy-2′-deoxyguanosine (8-OHdG); and antioxidant defense by superoxide dismutase (SOD), catalase (CAT), glutathione peroxidase (GPx), glutathione-related indices, and thiol/disulfide balance [13,14,15,16,17,18,19,20,21,22,23]. Their interpretation depends on sample type, healing phase, analytical method, local inflammation, systemic disease, and concordance with tissue-level outcomes.

Photobiomodulation (PBM) is increasingly interpreted as a controlled photochemical stimulus rather than as a nonspecific pro-healing or antioxidant intervention. Depending on wavelength, irradiance, fluence, exposure time, tissue penetration, treatment schedule, and disease context, red and near-infrared light can influence mitochondrial respiration, cytochrome c oxidase-related signaling, nitric oxide availability, adenosine triphosphate (ATP) generation, controlled ROS production, and downstream redox-sensitive pathways [24,25,26,27,28,29,30,31,32]. PBM is therefore best considered a biochemical redox-modulating intervention whose effects depend on dose, timing, and the pre-existing redox state of the target tissue.

Despite growing interest in PBM and oxidative stress biomarkers, findings remain difficult to compare across studies. Wound models, disease contexts, sample types, biomarker panels, irradiation parameters, treatment schedules, and outcome measures vary widely [5,32,33]. Many studies report wound closure without integrating histological repair quality, oxidative damage, antioxidant defense, and complete PBM dosimetry. Similarly, isolated markers such as MDA, TAC, or antioxidant enzyme activity cannot reliably distinguish adaptive ROS signaling from pathological oxidative injury outside the relevant biological and methodological context.

The main contribution of this review is a redox-guided interpretative framework for oral mucosal disease and repair. Rather than treating oral wound healing, oxidative stress, and PBM efficacy as separate topics, the review integrates biomarker interpretation, PBM dosimetry, healing phase, sample type, redox-sensitive biochemical pathways, and repair-quality outcomes. The framework follows the main pathways discussed later in the article, including nuclear factor erythroid 2-related factor 2/heme oxygenase-1 (Nrf2/HO-1), nuclear factor kappa B (NF-κB), hypoxia-inducible factor-1 alpha/vascular endothelial growth factor (HIF-1α/VEGF), mitogen-activated protein kinase/extracellular signal-regulated kinase (MAPK/ERK), phosphoinositide 3-kinase/protein kinase B (PI3K/Akt), and matrix metalloproteinase/tissue inhibitor of metalloproteinase (MMP/TIMP) signaling. This integrated perspective is intended to reduce marker-by-marker interpretation, place molecular mechanisms at the center of the discussion, and support rational PBM protocol development for oxidative-stress-related oral mucosal injury.

At the mechanistic level, this review is built on the premise that the source of ROS is as important as the magnitude of ROS production. Transient mitochondrial ROS and nitric oxide (NO)-related signals generated after appropriately dosed PBM may act as second messengers that support adaptive repair. In contrast, persistent nicotinamide adenine dinucleotide phosphate (NADPH) oxidase (NOX)-derived ROS in diabetes, infection, chronic inflammation, or mucositis may maintain NF-κB-dominated oxi-inflammatory injury. This source-specific interpretation provides the basis for linking PBM dosimetry, redox-sensitive pathways, biomarker selection, and repair-quality outcomes.

The review therefore follows a simple interpretive sequence: identify the redox source, place the biomarker in the correct healing phase and sample matrix, interpret it in relation to PBM dose and disease context, and confirm its biological meaning through repair-quality outcomes.

Table 1 summarizes the main conceptual gaps addressed by this review and clarifies how the proposed redox-guided framework differs from previous discussions of oral wound healing, oxidative stress, and photobiomodulation.

## 2. Review Strategy and Evidence Selection

This article was designed as a structured narrative review with thematic evidence mapping, rather than as a formal systematic review or meta-analysis. Its aim was to integrate mechanistic, biochemical, experimental, and translational evidence on oxidative-stress biomarkers in oral mucosal repair and photobiomodulation (PBM), with emphasis on redox-sensitive pathways, biomarker interpretation, PBM dosimetry, and repair-quality outcomes. The review was not intended to calculate pooled effect sizes, compare individual PBM protocols quantitatively, or provide formal clinical recommendations.

PubMed, Scopus, Web of Science, and Google Scholar were searched, with the final search performed on 25 May 2026. The search strategy combined established mechanistic literature with recent studies relevant to PBM dosimetry, redox signaling, salivary biomarkers, oral mucosal injury, translational wound models, molecular repair mechanisms, and methodological reporting standards.

The following search blocks guided retrieval: (1) (“oral wound healing” OR “oral mucosal wound” OR “oral mucosa” OR “oral mucositis”) AND (“oxidative stress” OR “redox signaling” OR “reactive oxygen species”); (2) (“photobiomodulation” OR “PBM” OR “low-level laser therapy” OR “LLLT”) AND (“oral wound healing” OR “oral mucositis” OR “oral mucosal repair”); (3) “photobiomodulation” AND (“oxidative stress” OR “redox signaling”) AND (“Nrf2” OR “HO-1” OR “NF-κB” OR “VEGF” OR “HIF-1α” OR “NOX” OR “MMP” OR “TIMP”); (4) “oral wound healing” AND (“TOS” OR “TAC” OR “OSI” OR “MDA” OR “4-HNE” OR “AOPP” OR “protein carbonyls” OR “8-OHdG” OR “SOD” OR “catalase” OR “glutathione peroxidase” OR “thiol/disulfide”); and (5) “salivary biomarkers” AND “oxidative stress” AND (“oral mucosa” OR “oral wound” OR “oral mucositis”).

Because older publications often use the term low-level laser therapy, both photobiomodulation and low-level laser therapy were included as search terms, although PBM is used as the preferred term in the text. Full-text English-language articles were prioritized. Non-English records were checked at the title, abstract, or metadata level and retained only when they provided unique historical or technical information. Conference abstracts, patents, editorials, and reports with insufficient methodological detail were not used as core evidence. Duplicate records were removed, and papers were excluded when the wound model, tissue context, irradiation conditions, or biomarker outcomes did not inform redox-based interpretation of oral mucosal repair.

Eligible literature was mapped to one or more thematic domains: oral mucosal repair; oxidative stress and redox signaling; PBM or low-level laser therapy; biomarkers of oxidant–antioxidant balance, lipid oxidation, protein oxidation, DNA oxidation, antioxidant defense, and thiol homeostasis; mitochondrial and NADPH oxidase-derived ROS; Nrf2/HO-1, NF-κB, HIF-1α/VEGF, MAPK/ERK, PI3K/Akt, and MMP/TIMP signaling; diabetes-associated wounds; therapy-related oral mucositis; salivary biomarker methodology; PBM dosimetry; and translational study design.

Priority was given to recent papers addressing current molecular mechanisms, PBM dose reporting, redox-sensitive pathways, saliva-based biomarkers, disease-specific models, or translational oral wound research. Older studies were retained when they established assays, redox concepts, wound-healing biology, PBM dose–response principles, or reporting standards that remain necessary for interpretation. Before finalizing the manuscript, the reference list was checked against these priorities, particularly recency, mechanistic relevance, methodological clarity, PBM dosimetry, biomarker interpretability, and applicability to oral mucosal repair.

Evidence was not ranked with a formal risk-of-bias tool; instead, it was interpreted qualitatively according to biological relevance, methodological transparency, recency, and translational value. Cell and ex vivo studies were used mainly to support cellular, mitochondrial, and pathway-level mechanisms. Animal studies were used to evaluate integrated repair biology, disease-context models, histology, angiogenesis, inflammation, extracellular matrix remodeling, and local or systemic redox outcomes. Clinical studies informed translational relevance, patient-centered outcomes, salivary biomarkers, and PBM reporting quality. Systematic reviews, consensus papers, and reporting guidelines were used to identify methodological standards and recurring gaps, while foundational assay papers supported interpretation of established redox methods.

Interpretation was considered stronger when similar patterns were supported across mechanistic studies, animal models, clinical observations, and reporting or consensus literature. Mechanisms demonstrated directly in oral mucosal wounds, oral mucositis, oral ulcers, oral cells, or oral PBM models were treated as primary evidence. Mechanisms supported mainly by related wound models, ex vivo systems, or general redox biology were treated as translationally supportive or biologically plausible rather than confirmed for oral mucosal PBM. Newer topics, including cysteine-specific redox switching, mitophagy, macrophage immunometabolism, inflammasome persistence, ferroptosis, extracellular-vesicle signaling, and omics-defined redox states, are therefore framed as hypotheses unless directly measured in oral mucosal PBM models.

This review is therefore not exhaustive and does not provide PRISMA screening, quantitative synthesis, or formal risk-of-bias scoring. Its purpose is to offer a transparent mechanistic synthesis that can guide biomarker selection, interpretation of PBM-related redox changes, and future oral mucosal wound-healing study design. The central organizing sequence is oral mucosal repair, redox imbalance, biomarker selection, PBM dose and context, molecular pathways, and repair-quality outcomes.

This evidence-mapping approach shaped the structure of the following biological sections. Literature on oral mucosal repair was used to establish the tissue and healing-phase context; redox biology and oxidative-stress biomarker studies were used to distinguish adaptive ROS signaling from persistent oxidative injury; PBM studies were used to connect light parameters with mitochondrial, nitric oxide-related, and redox-sensitive pathways; and translational wound-healing studies were used to link biomarker interpretation with repair-quality outcomes. The review therefore begins with the biological basis of oral mucosal repair before moving to ROS dynamics, disease-related oxidative damage, PBM mechanisms, biomarker selection, and translational study design.

To make the interpretative approach transparent, the evidence categories used throughout this review are summarized in Box 1.

Box 1 **Evidence interpretation framework used in this review.**

                                                            This review distinguishes four evidence categories:

 

Oral-specific PBM evidence: findings directly shown in oral mucosal wounds, oral mucositis, oral ulcers, gingival/oral cells, or oral PBM models; used as primary support for oral mucosal PBM interpretation.

 

Related wound-healing evidence: findings from skin wounds, diabetic wounds, inflammatory wounds, or non-oral mucosal repair models; used as translational support and clearly distinguished from oral-specific evidence.

 

General redox/mechanistic evidence: findings from mitochondrial, inflammatory, oxidative-stress, or cell-signaling studies; used to explain biological plausibility and pathway crosstalk.

 

Emerging hypotheses: mechanisms that are biologically plausible but not yet directly proven in oral mucosal PBM; presented as future research directions rather than established oral PBM mechanisms.

## 3. Biological Basis of Oral Mucosal Repair

The oral mucosa is a specialized barrier tissue continuously exposed to saliva, commensal and pathogenic microorganisms, mastication-related mechanical forces, dietary components, and immune surveillance. Compared with skin, oral wounds often re-epithelialize faster and form less prominent scars, reflecting distinct molecular and cellular programs within oral mucosal tissue [1,2,6]. This apparent efficiency should not be equated with simple surface closure. Effective repair also depends on epithelial migration, inflammatory regulation, extracellular matrix turnover, angiogenesis, microbial control, and restoration of mucosal barrier function [1,2,5,34].

Oral mucosal repair proceeds through overlapping phases of hemostasis, inflammation, proliferation, and remodeling. Immediately after injury, clot formation, platelet activation, and deposition of a provisional matrix create the first scaffold for cellular recruitment and tissue reconstruction. Neutrophils and monocytes/macrophages are then recruited to limit microbial invasion, remove damaged tissue, and regulate the local inflammatory environment [2,10,35]. This inflammatory response is necessary for repair, but it must remain controlled in both intensity and duration. If inflammation persists, epithelial migration may be impaired, matrix degradation may increase, and the transition toward proliferative repair may be delayed [10,35].

During the proliferative phase, epithelial cells migrate and expand from the wound margins, while fibroblasts support matrix deposition and wound contraction. Endothelial cells contribute to neovascularization and oxygen delivery, both of which are essential for tissue reconstruction [2,36,37]. Subsequent remodeling involves collagen maturation, matrix reorganization, and balanced activity of matrix metalloproteinases and tissue inhibitors of metalloproteinases [38,39]. In the oral cavity, these processes are further shaped by salivary proteins and antioxidants, resident microbiota, innate and adaptive immune activity, local mechanical forces, and systemic metabolic or inflammatory conditions [1,16,40,41,42].

Disease-related disruption of this coordinated repair program is particularly important in oxidative-stress-related conditions. Diabetes, chronic inflammation, infection, radiotherapy, chemotherapy, and chronic ulceration can shift the wound environment toward persistent inflammatory activation, mitochondrial dysfunction, vascular impairment, microbial dysbiosis, and oxidative damage to lipids, proteins, nucleic acids, and extracellular matrix components [3,4,35,43,44,45]. Under these conditions, oral mucosal repair is better understood as a redox-regulated biological process than as a simple sequence of closure events. This perspective supports evaluation of PBM not only through macroscopic wound closure, but also through redox-sensitive biochemical pathways, oxidative molecular damage, antioxidant recovery, histological repair quality, and restoration of barrier function [5,32].

Although this review focuses on the oral mucosa, many of the redox-sensitive mechanisms discussed here are not exclusive to the oral cavity. Similar principles of epithelial barrier disruption, immune-cell ROS production, mitochondrial redox signaling, NF-κB activation, Nrf2/HO-1-mediated cytoprotection, HIF-1α/VEGF-dependent vascular responses, MAPK/ERK and PI3K/Akt signaling, and MMP/TIMP-regulated extracellular matrix remodeling are also relevant to gastrointestinal, respiratory, and urogenital mucosal surfaces [46,47,48,49,50]. However, the oral mucosa has distinctive features that influence redox interpretation, including continuous salivary exposure, dense and dynamic microbial biofilms, mastication-related mechanical stress, frequent minor trauma, rapid epithelial turnover, site-specific epithelial architecture, and accessibility for both salivary biomarker assessment and local PBM application [1,16,40,41,42]. Thus, the pathways reviewed in this article should be viewed as broadly mucosal redox-repair mechanisms whose expression and translational interpretation are shaped by the specific oral environment.

## 4. Reactive Oxygen Species, Redox Signaling, and Photobiomodulation in Oral Wound Healing

### 4.1. Physiological ROS Production and Redox Signaling

Reactive oxygen species are continuously generated in aerobic tissues through mitochondrial respiration, NADPH oxidase activity, xanthine oxidase reactions, endoplasmic-reticulum redox processes, and inflammatory-cell enzyme systems [7,8,9]. Individual ROS differ substantially in half-life, reactivity, diffusion capacity, and molecular targets. Superoxide is short-lived and usually acts close to its site of production, whereas hydrogen peroxide is more stable and can function as a redox signal through reversible modification of redox-sensitive protein thiols [7,12]. Through these mechanisms, ROS help regulate cell survival, migration, proliferation, differentiation, inflammatory activation, and adaptive stress responses.

This context-dependent activity explains the distinction between oxidative eustress and oxidative distress. Oxidative eustress describes controlled oxidant signaling that remains compatible with antioxidant buffering and physiological regulation, whereas oxidative distress reflects excessive, sustained, or poorly compartmentalized ROS production that damages biomolecules and disrupts cell function [8,12]. Oral wound healing requires regulated ROS signaling for antimicrobial defense, keratinocyte migration, angiogenesis, macrophage activity, and coordination of repair. Delayed healing, by contrast, is associated with persistent ROS generation, inflammatory amplification, mitochondrial dysfunction, and oxidative molecular injury [10,11].

### 4.2. Oral Mucosal Redox Homeostasis

The oral mucosa has a dynamic redox microenvironment shaped by epithelial cells, immune cells, saliva, resident microbiota, dietary exposures, and mechanical forces. Basal ROS production is not inherently pathological, because regulated oxidant signaling contributes to epithelial surveillance, host–microbiome interaction, antimicrobial defense, and barrier homeostasis [1,41]. Saliva is particularly important in this setting. It contains antioxidant enzymes, glutathione-related systems, uric acid, thiols, electrolytes, antimicrobial proteins, and buffering components, and it can serve both as a modifier of the oral redox environment and as a clinically accessible source of redox biomarkers [16,40].

Salivary biomarkers are attractive for translational PBM studies, but their interpretation requires strict attention to sampling and processing conditions. The principal preanalytical sources of salivary variability are detailed in Section 8.3; here, it is sufficient to emphasize that salivary TOS, TAC, OSI, lipid-peroxidation products, protein-oxidation products, antioxidant enzymes, and thiol-related markers should be interpreted with oral disease status and systemic disease context [16,23,40,41].

### 4.3. ROS Dynamics Across Healing Phases

ROS production changes across the phases of wound repair. During early inflammation, platelet activation, neutrophil recruitment, monocyte infiltration, and macrophage activation generate oxidants that contribute to microbial control, debris clearance, and inflammatory signaling [10,11,35]. Myeloperoxidase (MPO)-derived oxidants and chlorinated protein products may be especially relevant during this phase, making MPO activity, AOPP, and TOS useful indicators of early inflammatory oxidative activity [51,52].

During the proliferative phase, moderate ROS signaling supports keratinocyte migration, fibroblast activity, angiogenesis, and extracellular matrix deposition. These effects are mediated in part through redox-sensitive pathways such as MAPK/ERK, PI3K/Akt, HIF-1α/VEGF, and Nrf2/HO-1 [36,37,53]. During remodeling, inflammatory ROS production should decline, antioxidant defense should recover, and extracellular matrix turnover should become more controlled. Persistent elevation of MDA, 4-HNE, protein carbonyls, AOPP, or 8-OHdG in later phases is therefore more consistent with unresolved oxidative injury than with adaptive signaling [13,18,19,20,52,54].

**Figure 1:**
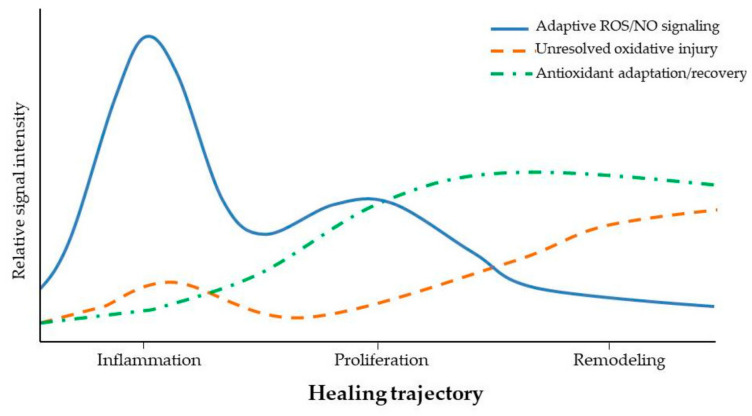
Phase-specific redox interpretation during oral mucosal wound healing. Adaptive ROS/NO signaling during the inflammatory phase supports host defense, microbial control, debris clearance, and repair initiation. During the proliferative phase, moderate redox signaling contributes to epithelial migration, fibroblast activity, angiogenesis, and extracellular matrix deposition, while antioxidant adaptation progressively increases to restore redox balance. Persistent elevation of oxidative damage markers, such as MDA/4-HNE, AOPP/protein carbonyls, or 8-OHdG, especially during proliferation or remodeling, is more consistent with unresolved oxidative injury than with adaptive signaling. The curves represent conceptual biomarker tendencies and should be interpreted together with sample matrix, disease context, PBM parameters, and tissue-level repair quality; they do not represent quantitative biomarker kinetics.

A conceptual phase-specific interpretation of redox biomarkers in oral mucosal repair is summarized in Figure 1.

### 4.4. Excessive ROS and Oxidative Damage in Disease-Related Delayed Healing

When oxidant generation exceeds antioxidant capacity, oxidative stress may impair epithelial renewal, fibroblast function, collagen synthesis, angiogenesis, mitochondrial metabolism, and inflammatory resolution [10,11,35]. Different oxidative stress biomarkers reflect different molecular targets and should not be treated as interchangeable measures. Lipid peroxidation can be assessed using MDA and 4-HNE, protein oxidation using protein carbonyls and AOPP, and oxidative DNA damage using 8-hydroxy-2′-deoxyguanosine [13,18,19,20,23,52,54].

Delayed oral mucosal healing is commonly associated with diabetes mellitus, chronic inflammation, infection, radiotherapy-associated mucosal injury, chemotherapy-induced oral mucositis, and chronic ulceration [3,4,11,43,44,45]. In diabetes, hyperglycemia promotes mitochondrial dysfunction, advanced glycation-end product formation, endothelial impairment, reduced nitric oxide bioavailability, and excessive ROS generation [4,55]. In oral mucositis, epithelial injury, inflammatory cytokine amplification, mitochondrial dysfunction, oxidative stress, and DNA damage contribute to mucosal breakdown and delayed recovery [3,43,44,45]. Because these conditions differ in their dominant redox and inflammatory disturbances, PBM-related biomarker changes should be interpreted according to disease context rather than as uniform antioxidant effects.

Recurrent aphthous stomatitis (RAS) represents another clinically relevant oral mucosal condition in which the redox-guided framework may be useful. RAS is characterized by recurrent painful ulceration of the non-keratinized oral mucosa and has a multifactorial pathogenesis involving epithelial barrier disruption, immune dysregulation, genetic susceptibility, local trauma, microbial factors, nutritional deficiencies, stress-related factors, and inflammatory activation [56,57,58]. Although the precise cause of RAS remains incompletely understood, several studies and meta-analyses indicate that patients with RAS may show increased oxidative stress and reduced antioxidant defense in saliva and/or blood, including changes in markers such as MDA, TAC/TAS, SOD, GPx, and related oxidant–antioxidant indices [57,58,59]. In this context, recurrent ulceration may reflect repeated cycles of mucosal injury, inflammatory-cell activation, ROS generation, epithelial breakdown, and incomplete restoration of redox and barrier homeostasis. The pathways discussed in this review, particularly NF-κB-mediated inflammatory signaling, Nrf2/HO-1-associated cytoprotection, mitochondrial ROS signaling, antioxidant defense, VEGF-related vascular responses, and MMP/TIMP-regulated matrix remodeling, may therefore be relevant to RAS pathogenesis and lesion progression. However, pathway-level evidence in RAS remains less developed than clinical and biomarker evidence, and future studies should integrate salivary or tissue redox biomarkers with inflammatory mediators, epithelial repair markers, and PBM parameters. PBM has been investigated for pain reduction and acceleration of healing in RAS, but its redox mechanisms in this condition remain insufficiently defined [60,61,62]. Therefore, RAS may serve as a clinically accessible model for testing whether PBM modulates not only pain and ulcer duration, but also redox balance, inflammatory resolution, and mucosal repair quality. The main disease-context considerations for redox-guided PBM interpretation are summarized in Box 2.

Box 2 **Disease-context framing for redox-guided PBM in oral mucosal repair.**

Diabetes-associated oral wounds: persistent oxidant burden, endothelial dysfunction, impaired NO signaling, mitochondrial stress, and delayed epithelial repair [4,55,63,64,65]. Useful markers include TOS, TAC, OSI, MDA/4-HNE, AOPP/protein carbonyls, SOD/CAT/GPx, glutathione (GSH) or thiol/disulfide balance, Nrf2/HO-1, NOX enzymes, and VEGF.

 

Chemotherapy- or radiotherapy-induced oral mucositis: epithelial injury, inflammatory cytokine amplification, mitochondrial dysfunction, oxidative DNA damage, apoptosis or senescence, and barrier disruption [3,43,44,45]. Useful markers include 8-OHdG, MDA/4-HNE, protein carbonyls, NF-κB, Nrf2/HO-1, VEGF, and inflammatory mediators.

 

Chronic inflammation, infection, and ulceration: neutrophil/macrophage activation, MPO-related oxidant production, dysbiosis, persistent NF-κB signaling, matrix degradation, and impaired remodeling [38,51,66,67,68,69]. Useful markers include MPO-related activity, AOPP, protein carbonyls, TOS/OSI, MMP/TIMP balance, NOX enzymes, and salivary inflammatory markers.

Recurrent aphthous stomatitis: recurrent epithelial ulceration, immune-inflammatory activation, possible salivary and systemic oxidant–antioxidant imbalance, and repeated disruption of mucosal barrier repair [56,57,58,59]. Useful markers include salivary and/or serum MDA, TAC/TAS, TOS/OSI, SOD, GPx, thiol/disulfide balance, inflammatory mediators, epithelial repair markers, and, in future mechanistic studies, NF-κB, Nrf2/HO-1, VEGF, and MMP/TIMP-related markers.

 

Across these disease contexts, PBM should be evaluated by whether it restores a redox state compatible with repair, not by whether it simply lowers every oxidant-related marker.

Together, these disease contexts support the use of oral mucosal repair as a biochemical model of oxidative stress, inflammation resolution, and tissue regeneration.

### 4.5. Redox-Sensitive Biochemical Pathways

Redox-sensitive pathways provide the mechanistic link between biomarker changes and repair outcomes, but they must be interpreted through pathway crosstalk rather than isolated marker lists. PBM can influence mitochondrial respiration and nitric oxide availability through mitochondrial photoacceptors, including cytochrome c oxidase and additional non-mitochondrial targets [24,25,27,28,30,63]. When the delivered dose remains within a repair-compatible range, low-level mitochondrial ROS may act as second messengers that reversibly modify redox-sensitive protein thiols, thereby supporting MAPK/ERK, PI3K/Akt, Nrf2/HO-1, and HIF-1α/VEGF signaling [7,8,9,10,37,53,64,65,66,67,68].

This mitochondrial signaling must be distinguished from sustained NOX-derived ROS. NOX activity is useful during early host defense and inflammatory-cell coordination, but persistent NOX-driven oxidant generation in diabetes, infection, chronic inflammation, or mucositis may maintain NF-κB activation, increase cytokine production, and promote protein, lipid, DNA, and matrix damage [69,70,71,72]. Accordingly, the biological meaning of a ROS-related biomarker depends not only on its concentration, but also on its cellular source, timing, and relationship with inflammatory resolution and tissue remodeling.

The redox-sensitive pathways discussed in oral mucosal repair are shared signaling modules rather than wound-specific mechanisms. NF-κB, Nrf2/HO-1, HIF-1α/VEGF, MAPK/ERK, PI3K/Akt, NOX-derived ROS signaling, and MMP/TIMP-regulated extracellular matrix remodeling also participate in chronic inflammation, tumor development, and cancer progression [73,74,75,76,77,78]. Their biological meaning depends on the duration, source, intensity, and compartmentalization of ROS production. During physiological repair, transient mitochondrial ROS and nitric oxide-related signaling may support epithelial migration, inflammatory-cell coordination, angiogenesis, fibroblast activity, and extracellular matrix remodeling. In contrast, persistent NOX-derived or mitochondria-derived ROS in chronic inflammation may maintain NF-κB activation, cytokine amplification, oxidative DNA damage, protein and lipid oxidation, and matrix degradation [73,74,75]. In tumor-related contexts, the same pathways may support genomic instability, hypoxia adaptation, VEGF-mediated angiogenesis, epithelial–stromal interactions, invasion, and remodeling of the tumor microenvironment [74,76,77,78]. Thus, the distinction between adaptive redox signaling and pathological oxidative stress extends beyond wound healing, but PBM-related interpretation should remain context-specific because a repair-compatible response in an acute wound may have different implications in chronically inflamed, dysplastic, or neoplastic tissue.

A favorable PBM response may involve coordinated pathway switching rather than uniform oxidant suppression. Early, controlled NF-κB activity may support defense and repair initiation, whereas prolonged NF-κB dominance with elevated AOPP, protein carbonyls, MDA/4-HNE, or 8-OHdG suggests unresolved oxi-inflammatory injury. In contrast, Nrf2/HO-1 activation may increase glutathione-related enzymes, thioredoxin systems, and antioxidant buffering, thereby limiting molecular damage while preserving the redox signaling required for epithelial migration and fibroblast function [64,65,79].

In the pathway model proposed here, PBM within an appropriate dose range first influences mitochondrial and membrane-associated photoacceptor systems, leading to changes in electron transport, NO availability, ATP support, calcium-linked signaling, and a transient hydrogen peroxide signal. This signal may then be translated into cellular responses through reversible cysteine/thiol modifications of redox-sensitive kinases, phosphatases, and transcriptional regulators. Better-supported components of this model include mitochondrial and NO-related PBM signaling, NF-κB-linked inflammatory activation, and Nrf2/HO-1-supported cytoprotection. The inferred component is that PBM-driven transient mitochondrial ROS helps shift the balance from persistent NF-κB/NOX activity toward Nrf2-associated antioxidant adaptation in oral mucosal repair. Still unproven in oral mucosal PBM models are the exact cysteine targets, the cell-specific sequence of pathway activation, the causal hierarchy between Nrf2 and NF-κB, and the PBM parameter thresholds that separate adaptive redox signaling from oxidative injury [7,8,9,10,24,25,29,64,65,66,79,80].

Downstream repair quality depends on convergence between redox signaling, angiogenesis, and extracellular matrix remodeling. HIF-1α/VEGF links oxygen sensing and redox biology with neovascularization; MAPK/ERK and PI3K/Akt support keratinocyte migration, fibroblast survival, proliferation, and antioxidant adaptation; and ROS-regulated MMP/TIMP balance controls collagen turnover and mucosal architecture [37,38,39,53,66,81,82]. Interpreting PBM through these interacting pathways moves the analysis beyond descriptive oxidative stress measurement toward a mechanistic model that connects dose, redox source, pathway activation, and repair phenotype.

Together, these pathway interactions define PBM as a dose- and context-dependent redox-modulating stimulus. In impaired oral mucosal repair, the key question is not whether PBM lowers every oxidant-related marker, but whether it shifts the wound environment toward controlled mitochondrial signaling, inflammatory resolution, extracellular matrix organization, and tissue reconstruction.

### 4.6. PBM Dose, ROS Signaling, and the Redox Therapeutic Window

PBM commonly follows a biphasic dose–response pattern. Insufficient exposure may produce little biological effect, an appropriate exposure range may support mitochondrial, nitric oxide, and adaptive ROS signaling, and excessive exposure may become ineffective, inhibitory, or pro-oxidant [83,84,85,86]. This response depends on wavelength, fluence, irradiance, exposure time, pulse mode, spot size, treatment schedule, tissue optical properties, oxygen availability, and baseline redox state [32,33,83,84,85,86,87].

In this review, the redox therapeutic window is defined as the range of PBM parameters that generates sufficient transient ROS/NO and metabolic signaling to activate adaptive repair pathways while avoiding sustained oxidative distress, inflammatory amplification, or inhibition of cell migration and proliferation. This window is not a fixed dose interval; it is expected to shift according to tissue depth, optical properties, oxygen availability, wound stage, inflammatory burden, diabetes or mucositis status, and baseline antioxidant reserve.

**Figure 2:**
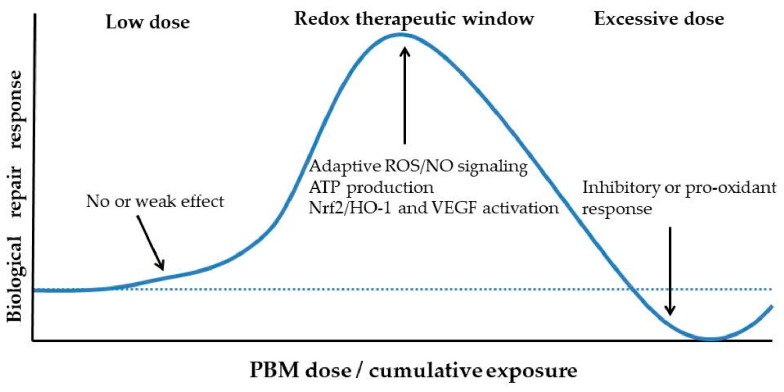
PBM dose–response and redox therapeutic window in oral mucosal repair. Low PBM exposure may be biologically insufficient, an appropriate exposure range may support ATP production, nitric oxide availability, adaptive ROS signaling, Nrf2/HO-1 activation, angiogenesis, and repair, whereas excessive exposure may become inhibitory or pro-oxidant. The therapeutic window is context-dependent and should be interpreted according to complete dosimetry, tissue optical properties, oxygen availability, wound phenotype, baseline redox state, and integrated redox, histological, molecular, and clinical outcomes. The curve is a conceptual schematic and should not be interpreted as a fixed dose equation or universal PBM threshold.

The dose-dependent relationship between PBM and redox signaling is illustrated in Figure 2.

The following subsection extends this classical PBM-redox model to emerging mechanisms that may help explain why similar PBM protocols can produce different responses across oral wound phenotypes.

### 4.7. Emerging and Extrapolated Molecular Mechanisms in Redox-Guided Photobiomodulation

Although these mechanisms are increasingly relevant to redox biology and wound repair, their confirmation in oral mucosal PBM models remains uneven. They are therefore discussed according to evidence strength: mechanisms directly demonstrated in oral PBM models are treated as primary evidence, mechanisms supported by related wound or mucosal models are considered translationally supportive, and mechanisms derived mainly from general redox biology are presented as biologically plausible hypotheses for future studies.

Recent PBM research has moved beyond the classical view that red and near-infrared light act only through cytochrome c oxidase (CCO). PBM is better interpreted as a multi-target photobiological stimulus that can influence mitochondrial respiration, NO signaling, membrane-associated receptors and ion channels, calcium-dependent pathways, redox-sensitive transcription factors, extracellular-vesicle communication, and tissue-specific repair networks [24,25,27,30,87,88,89,90,91].

Redox-sensitive cysteine/thiol switches are an important molecular layer for interpreting PBM. Hydrogen peroxide and related oxidants can act as signaling molecules when they reversibly modify protein thiols through cysteine oxidation, S-glutathionylation, disulfide formation, and related redox post-translational modifications. In this context, PBM-induced transient mitochondrial ROS should not be judged only by total oxidant concentration. Its biological relevance also depends on whether it regulates redox-sensitive kinases, phosphatases, transcriptional regulators, and cytoskeletal or matrix-related proteins that control migration, proliferation, inflammation, and antioxidant adaptation [7,8,9,10,17,66,80].

Mitochondrial dynamics and mitochondrial quality control are another emerging area. PBM may influence mitochondrial membrane potential, electron transport, ATP generation, NO availability, and controlled ROS production, but mitochondrial function is also shaped by fission/fusion balance, biogenesis, and mitophagy, defined as the selective autophagic removal of damaged or dysfunctional mitochondria [24,25,27,28,63,88,92]. In delayed oral mucosal repair, persistent mitochondrial injury could amplify ROS production, DNA damage, inflammasome activation, apoptosis, senescence, or impaired epithelial migration. In this context, inflammasome activation refers to stimulation of cytosolic danger-sensing protein complexes that promote inflammatory cytokine maturation and may prolong tissue injury. Future PBM-redox studies should consider markers of mitochondrial quality control, such as PTEN-induced kinase 1 (PINK1)/Parkin-related mitophagy, microtubule-associated protein 1 light chain 3 (LC3)/p62 autophagic flux, dynamin-related protein 1 (DRP1)/mitofusin (MFN)-related dynamics, mitochondrial membrane potential, and mitochondrial DNA damage, when the study design allows.

Macrophage polarization and immunometabolism provide a bridge between redox signaling and inflammation resolution. Oral wound healing depends on the timely transition from early antimicrobial and pro-inflammatory macrophage activity toward pro-resolving and reparative phenotypes, a process influenced by oxygen availability, glycolysis, oxidative phosphorylation, NO metabolism, cytokine signaling, and microbial cues [34,35]. PBM may affect this transition indirectly through mitochondrial and redox-sensitive signaling, but oral mucosal PBM studies rarely define macrophage phenotype, metabolic state, and redox pathway activation in the same model. Future studies should combine redox biomarkers with macrophage markers, such as inducible nitric oxide synthase (iNOS), cluster of differentiation 86 (CD86), arginase-1, cluster of differentiation 206 (CD206), interleukin-1β (IL-1β), interleukin-10 (IL-10), and transforming growth factor beta (TGF-β), together with tissue-level repair outcomes.

Inflammasome activation should also be integrated into the redox model. In diabetic or chronically inflamed wounds, NF-κB priming, mitochondrial dysfunction, ROS accumulation, and danger-associated signals can activate the NOD-like receptor family pyrin domain-containing 3 (NLRP3) inflammasome. NF-κB is a redox-sensitive inflammatory transcription factor that promotes expression of cytokines and inflammasome-related components, whereas the NLRP3 inflammasome is a cytosolic danger-sensing complex that contributes to maturation of interleukin-1β (IL-1β) and interleukin-18 (IL-18). Persistent activation of this axis may promote pyroptotic cell stress, sustained inflammation, and delayed repair [93,94]. For PBM studies, the key unresolved question is whether appropriately dosed light can reduce pathological NF-κB/NLRP3 persistence while preserving the early inflammatory signals required for microbial control and repair initiation. This distinction is particularly relevant in oral mucositis, diabetes-associated oral wounds, and infected or dysbiotic mucosal lesions.

Ferroptosis adds a lipid-centered dimension to oxidative mucosal injury. Ferroptosis is an iron-dependent form of regulated cell death driven by lipid-peroxide accumulation and insufficient lipid-peroxide repair. Therefore, MDA or 4-HNE alone cannot distinguish general lipid oxidation from ferroptotic cell death [18,19,95,96]. In studies of severe mucosal injury, diabetes, radiotherapy, chemotherapy, or chronic inflammation, PBM-related lipid-peroxidation outcomes could be strengthened by including glutathione peroxidase 4 (GPX4), solute carrier family 7 member 11/cystine-glutamate antiporter (SLC7A11/xCT), acyl-CoA synthetase long-chain family member 4 (ACSL4), ferritin/iron indices, glutathione status, and lipid ROS-sensitive readouts. At present, ferroptosis remains a plausible but insufficiently tested mechanism in oral mucosal PBM.

Extracellular vesicles (EVs) and systems-level profiling may help connect local redox events with multicellular tissue responses. EVs released by epithelial cells, fibroblasts, endothelial cells, immune cells, platelets, or mesenchymal stromal cells can carry proteins, lipids, microRNAs (miRNAs), and redox-related cargo that influence inflammation, angiogenesis, fibroblast activity, and matrix remodeling [87,91]. PBM has been reported to affect EV secretion in some experimental contexts, but the relevance of PBM-modulated EV cargo to oral mucosal redox repair remains largely unexplored [87].

Finally, omics-based approaches could move the field beyond candidate markers. Transcriptomics, proteomics, metabolomics, redox proteomics, single-cell RNA sequencing, and spatial profiling can identify cell-specific redox states, macrophage or fibroblast subpopulations, epithelial stress programs, mitochondrial signatures, cysteine oxidation patterns, and pathway networks that cannot be resolved by TOS, TAC, MDA, or SOD alone [31,80,97]. These approaches are especially useful for determining whether PBM produces coordinated pathway remodeling rather than isolated biomarker changes.

Overall, the strongest evidence supports PBM effects on mitochondrial activity, nitric oxide signaling, controlled ROS generation, inflammatory modulation, and selected repair pathways. More tentative, but biologically plausible, are PBM effects on cysteine-specific redox switches, mitophagy, macrophage immunometabolism, inflammasome persistence, ferroptosis, and EV-mediated intercellular redox communication in oral mucosal wounds. These mechanisms should be presented as testable hypotheses unless they are directly measured in oral mucosal PBM models.

**Figure 3:**
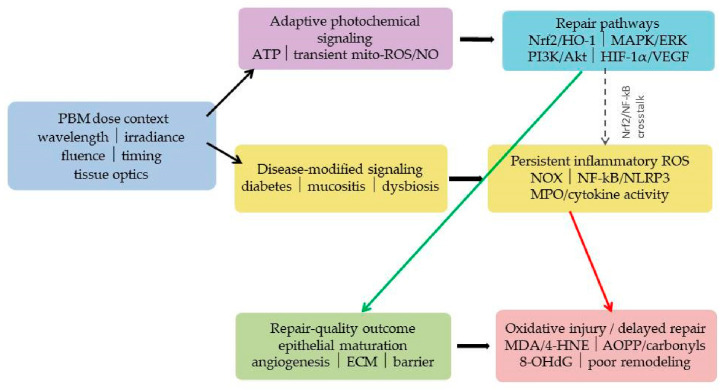
Mechanistic crosstalk in redox-guided photobiomodulation of oral mucosal repair. PBM may influence mitochondrial photoacceptors, nitric oxide availability, ATP support, and controlled mitochondrial ROS signaling. These signals are placed in relation to NOX-derived inflammatory ROS, Nrf2/HO-1 cytoprotection, NF-κB activity, HIF-1α/VEGF-mediated angiogenesis, MAPK/ERK and PI3K/Akt-mediated epithelial and fibroblast responses, and MMP/TIMP-regulated extracellular matrix remodeling. The figure emphasizes that PBM responses should be interpreted as phase- and context-dependent recalibration of redox signaling rather than as nonspecific ROS suppression. The diagram is a conceptual pathway map rather than a validated causal or quantitative model.

The mechanistic crosstalk that should guide PBM-redox interpretation is summarized in Figure 3.

### 4.8. Minimal Interpretive Rule for PBM-Redox Biomarker Evaluation

For PBM studies, a redox biomarker should be interpreted only after four questions are answered: What sample matrix was used? Which healing phase was assessed? What PBM dose and schedule were applied? Does the biomarker change correspond to repair-quality outcomes? This minimal rule prevents isolated changes in TOS, TAC, MDA, SOD, or related markers from being overinterpreted as either beneficial antioxidant effects or harmful oxidative stress [13,14,15,16,17,18,19,20,21,22,23,28,52,54,64,65,66,69,70,71,72,98]. Interpretation should also follow the evidence framework above by separating oral-specific PBM findings from related wound evidence, general redox mechanisms, and emerging hypotheses.

**Figure 4:**
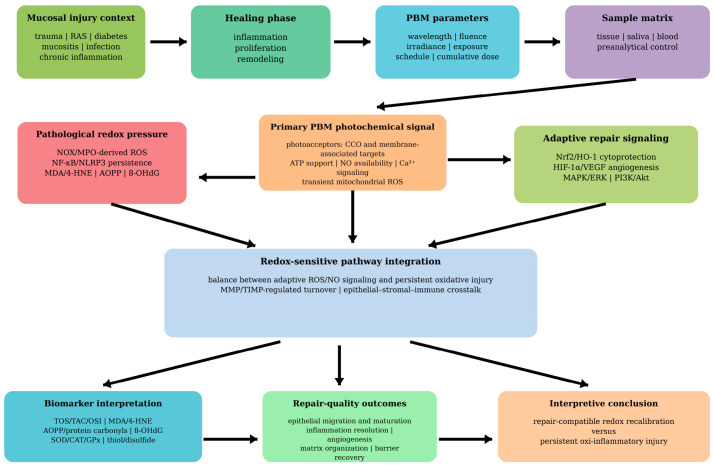
Redox-guided photobiomodulation in oral mucosal repair. The schematic integrates mucosal injury context, healing phase, PBM parameters, sample matrix, primary PBM photochemical signaling, pathological redox pressure, adaptive repair signaling, redox-sensitive pathway integration, biomarker interpretation, and repair-quality outcomes. It emphasizes the distinction between adaptive ROS/NO signaling and persistent oxi-inflammatory injury and links PBM-related responses with Nrf2/HO-1 cytoprotection, HIF-1α/VEGF-mediated angiogenesis, MAPK/ERK and PI3K/Akt signaling, NOX/MPO-derived ROS, NF-κB/NLRP3 persistence, MMP/TIMP-regulated matrix turnover, epithelial repair, inflammation resolution, angiogenesis, and mucosal barrier recovery. The figure is a conceptual schematic intended to support study design and interpretation; it is not a validated diagnostic, prognostic, or therapeutic algorithm.

Together, these considerations support a dedicated schematic overview of redox-guided photobiomodulation in oral mucosal repair. The proposed framework, integrating mucosal injury context, healing phase, PBM parameters, sample matrix, primary PBM photochemical signaling, pathological redox pressure, adaptive repair signaling, biomarker interpretation, and repair-quality outcomes, is summarized in Figure 4.

Having defined the redox sources, disease contexts, and PBM-sensitive pathways, the following section focuses on biomarkers as interpretive tools. Markers are therefore discussed according to the biological information they add, rather than by restating the general role of ROS in repair.

## 5. Biomarkers of Oxidative Balance and Molecular Damage

### 5.1. Principles of Biomarker Selection

Biomarker selection in oral wound-healing studies should start from the biological question, not from the easiest available assay. Because ROS can support normal signaling as well as cause molecular injury, informative panels should combine global redox balance with markers of damage, antioxidant defense, and, where relevant, pathway activation [7,9,13,16,23,40,99]. In PBM studies, this distinction is particularly important because the expected response is not blanket ROS removal, but re-establishment of a redox environment that allows inflammation to resolve and tissue structure to recover.

Sampling time is equally important. Oxidant activity early after injury may represent host defense or inflammatory signaling, whereas persistence of damage markers during proliferation or remodeling more often indicates failed resolution. For this reason, biomarker results should be read together with wound phase, sample matrix, assay method, PBM dose, disease context, and tissue-level outcomes rather than as isolated proof of benefit or harm [23,32,99,100,101].

### 5.2. Integrated Markers of Redox Balance

TOS, TAC, and OSI help describe the broad direction of oxidant–antioxidant change. TOS captures cumulative oxidizing activity, TAC reflects the combined buffering potential of antioxidants, and OSI expresses the balance between oxidant load and antioxidant reserve [14,15]. They are useful for comparing groups, time points, disease states, or PBM protocols, but they do not identify the oxidant source, specific antioxidant pathway, cellular compartment, or molecular target involved.

These integrated indices are best interpreted as entry-level redox readouts rather than mechanism-specific biomarkers. A fall in TOS or OSI may suggest a lower overall oxidant burden, but it cannot show whether this reflects reduced NOX activity, improved mitochondrial coupling, weaker neutrophil/MPO activity, salivary-flow effects, or matrix-related assay interference. Likewise, an increase in TAC may indicate restored antioxidant reserve without identifying the responsible antioxidant system or showing whether useful transient ROS signaling has been preserved. When mechanistic interpretation is needed, integrated indices should be combined with molecular damage markers, antioxidant enzymes or thiol/disulfide status, and pathway-specific markers [13,14,15,16,17,23,99].

In oral wound research, tissue, blood, and saliva provide different windows into redox biology. Tissue is closest to the wound microenvironment, blood describes systemic redox status, and saliva offers a practical non-invasive matrix for oral monitoring. Salivary results should follow the standardized collection and processing principles summarized in Section 8.3 rather than being interpreted as direct surrogates of tissue redox status. Thus, TOS, TAC, and OSI are most informative when evaluated with histology, inflammation, angiogenesis, epithelial repair, and overall repair quality.

### 5.3. Lipid Peroxidation Markers

Lipid peroxidation markers indicate oxidative changes in membrane lipids and lipid-derived signaling molecules. MDA is frequently measured because the assay is accessible and comparable across many studies, but TBARS-based methods are not specific for MDA and may also detect other reactive compounds [18,102]. 4-HNE adds complementary information because it is both a lipid peroxidation product and a signaling-active aldehyde that can modify proteins and alter cellular pathways [19].

In delayed oral healing, excessive lipid peroxidation may impair membrane integrity, mitochondrial function, epithelial migration, fibroblast activity, and inflammatory resolution. In PBM studies, reduced MDA or 4-HNE supports a beneficial redox interpretation only when it is accompanied by improved structural repair, antioxidant recovery, and adequately reported irradiation parameters [32]. Lipid peroxidation markers are therefore most useful as part of a broader repair profile, not as isolated proof of therapeutic success.

### 5.4. Protein Oxidation Markers

Protein oxidation markers capture oxidative modification of enzymes, structural proteins, and signaling mediators. Protein carbonyls provide a broad readout of oxidative protein damage, while AOPP are more closely linked to chlorinated oxidants and myeloperoxidase-related inflammatory activity [20,52]. These markers are relevant because oxidative changes in matrix proteins, receptors, cytokines, and enzymes can disturb epithelial regeneration, fibroblast activity, collagen organization, and matrix remodeling.

AOPP and protein carbonyls complement lipid peroxidation markers and are particularly informative in models characterized by persistent inflammation or neutrophil-dominated oxidative activity. Their combined assessment can help distinguish membrane-centered lipid injury from protein-centered oxidative damage and reduce overreliance on a single endpoint.

### 5.5. Oxidative DNA Damage Markers

8-OHdG is commonly used to assess oxidative guanine modification and may reflect nuclear or mitochondrial DNA injury [54]. It is especially relevant in chronic inflammation, mitochondrial dysfunction, radiotherapy-associated injury, chemotherapy-induced oral mucositis, diabetes, and other delayed-healing states in which oxidative damage may affect epithelial renewal, apoptosis, senescence, and tissue recovery [3,43,44,45].

The biological meaning of 8-OHdG depends on wound stage, cell type, tissue compartment, and associated inflammatory activity. A transient increase during acute inflammation may not have the same implication as persistent elevation during the proliferative or remodeling phases. In PBM research, 8-OHdG may be useful for evaluating repair quality and safety, particularly when interpreted alongside proliferation, apoptosis, inflammation, oxidative damage, and wound-closure outcomes.

### 5.6. Antioxidant Defense Markers

Antioxidant defense markers describe how tissues or fluids buffer oxidant activity. SOD dismutates superoxide to hydrogen peroxide, whereas catalase and GPx participate in hydrogen peroxide detoxification. Glutathione-related systems and thiol/disulfide balance add information on intracellular and extracellular redox buffering [17,21,22].

These markers are adaptive and phase-dependent. Increased antioxidant enzyme activity may indicate protective activation, whereas reduced activity in the presence of elevated damage markers may indicate antioxidant exhaustion, enzyme inhibition, or delayed recovery. PBM may restore TAC, thiol balance, or antioxidant enzyme activity, but these changes should be interpreted in relation to oxidant burden, molecular damage, sampling time, PBM dose, and tissue repair outcomes.

### 5.7. Mechanistic and Pathway-Related Biomarkers

Pathway-related biomarkers connect redox measurements with mechanisms of repair. NOX enzymes identify regulated enzymatic ROS sources; Nrf2/HO-1 indicates antioxidant and cytoprotective signaling; NF-κB reflects inflammatory activation; HIF-1α/VEGF links redox biology with hypoxia adaptation and angiogenesis; nitrite/nitrate provide indirect information on nitric oxide metabolism; and MMP/TIMP markers assess extracellular matrix turnover [28,37,65,69,72].

In PBM studies, these markers are especially important because they help distinguish redox modulation from nonspecific antioxidant effects. For example, reduced oxidative damage together with increased or restored Nrf2/HO-1 activity supports adaptive cytoprotection, whereas persistent NF-κB activation with elevated AOPP or protein carbonyls suggests unresolved oxi-inflammatory activity. Similarly, changes in VEGF or MMP/TIMP balance may help determine whether redox modulation is accompanied by improved angiogenesis and matrix remodeling.

### 5.8. Practical Biomarker Panels for Experimental and Translational Studies

A practical biomarker panel should be selected according to the model, sample type, and biological question. Tissue homogenates are most appropriate for local oxidative damage, antioxidant defense, inflammation, angiogenesis, and pathway activation. Saliva is more feasible for non-invasive clinical monitoring but requires strict preanalytical control. Blood is useful when systemic redox status is relevant, although it should not be treated as a direct substitute for the oral wound microenvironment [16,23,40,99].

For PBM-oriented oral wound studies, a minimal panel should include one global redox marker, one molecular damage marker, one antioxidant defense marker, and at least one tissue-level repair outcome. Extended panels can add 8-OHdG, thiol/disulfide balance, Nrf2/HO-1, NF-κB, VEGF, NOX, nitrite/nitrate, and MMP/TIMP markers when these variables match the wound phase and disease context. The aim is not to maximize the number of markers, but to combine complementary endpoints that distinguish persistent oxidative injury from adaptive redox signaling.

MDA alone, TAC alone, or isolated antioxidant enzyme activity cannot determine whether PBM reduces molecular damage, restores antioxidant reserve, improves inflammatory or mitochondrial signaling, or supports repair quality. Biomarker selection should remain hypothesis-driven and explicitly linked to PBM parameters, sampling conditions, wound phase, disease phenotype, and histological or clinical outcomes.

### 5.9. Critical Appraisal of Biomarker Strengths and Limitations

Oxidative stress biomarkers do not all carry the same interpretative weight. TOS, TAC, and OSI describe the overall direction of redox imbalance but do not identify oxidant sources or molecular targets. MDA remains a common lipid peroxidation marker, although TBARS-based methods have specificity limitations [18,102]. In contrast, 4-HNE can add more mechanistic information because it is both a lipid peroxidation product and a bioactive aldehyde [19].

Protein carbonyls provide a broad measure of oxidative protein damage, whereas AOPP may be particularly informative in neutrophil-rich or MPO-associated inflammation [20,52]. 8-OHdG is useful when oxidative DNA injury, apoptosis, senescence, mucositis, radiation injury, or chronic inflammation are central to the model, but it should not be treated as a general marker of wound closure [54]. Antioxidant enzymes such as SOD, CAT, and GPx are also context-dependent: increased activity may reflect adaptive protection, whereas reduced activity may indicate exhaustion, inhibition, or delayed recovery [21,22].

Sample type further affects interpretation. Tissue provides the most direct local information on wound redox biology, saliva is more realistic for non-invasive clinical translation, and blood is most useful when systemic disease contributes to wound impairment. For salivary measurements, the standardized collection and processing requirements summarized in Section 8.3 should be applied before comparing biomarker values across groups, time points, or PBM protocols. The most robust approach is therefore a biologically justified panel that combines global redox status, molecular damage, antioxidant defense, pathway activation, and tissue-level repair quality.

The interpretative strengths and limitations of the main oxidative stress biomarker groups are summarized in Table 2, while practical minimal and extended biomarker panels for different PBM-oriented study aims are presented in Table 3.

## 6. Experimental Models and Study Designs

### 6.1. Rationale for Model Selection

Experimental models should be selected according to the biological question. Cell-based systems are most appropriate for examining cellular mechanisms, redox-sensitive signaling, mitochondrial responses, and PBM parameter screening. Three-dimensional and ex vivo models add tissue architecture and barrier properties, while animal models provide integrated repair biology, including inflammation, vascular response, extracellular matrix remodeling, and systemic metabolic influences. Clinical studies are ultimately required to determine translational relevance in heterogeneous patient populations [5,36].

For redox-oriented PBM research, model selection should not be based only on convenience or the speed of wound closure. Robust designs integrate macroscopic wound area, re-epithelialization, inflammatory activity, angiogenesis, extracellular matrix organization, oxidative damage, antioxidant defense, pathway activation, and complete PBM dosimetry [5,32]. Faster closure should not be interpreted as complete repair if it occurs alongside persistent oxidative damage, poor matrix organization, inadequate epithelial maturation, or unresolved inflammation.

### 6.2. In Vitro Cell-Based Models

In vitro models using oral keratinocytes, gingival fibroblasts, periodontal ligament fibroblasts, endothelial cells, macrophages, or co-cultures are useful for studying viability, migration, proliferation, mitochondrial activity, inflammatory mediator release, antioxidant responses, and redox-sensitive signaling [26,36,53]. Scratch assays can provide standardized information on cell migration, while oxidative or inflammatory stress can be induced using hydrogen peroxide, high glucose, cytokines, bacterial components, or mitochondrial stressors.

The main advantage of in vitro models is experimental control. PBM parameters such as wavelength, irradiance, fluence, exposure time, pulse mode, number of sessions, and irradiation timing can be adjusted precisely. However, conventional monolayer cultures do not reproduce saliva, microbiome interactions, vascular supply, systemic metabolism, immune-cell trafficking, or tissue-level mechanical forces. In vitro results should be interpreted as mechanistic signals that require validation in three-dimensional, animal, or clinical models.

### 6.3. Three-Dimensional and Ex Vivo Models

Three-dimensional and ex vivo systems provide an intermediate level of biological complexity between monolayer cell culture and in vivo experiments. Organotypic oral mucosal equivalents, reconstructed epithelial models, spheroids, hydrogel-based systems, and tissue explants better reproduce epithelial stratification, cell–matrix interaction, oxygen and nutrient gradients, and tissue-like barrier responses [2,5,103]. These models are particularly useful for evaluating epithelial barrier restoration, oxidative injury, inflammatory mediator diffusion, extracellular matrix organization, and PBM penetration.

These models may also clarify whether PBM effects differ between superficial epithelial layers and deeper stromal compartments. This distinction is important because light distribution depends on wavelength, tissue thickness, hydration, pigmentation, scattering, absorption, beam geometry, and source-to-tissue distance [32,33]. Nevertheless, these models have limited experimental duration and cannot fully reproduce systemic immune, vascular, endocrine, neural, or metabolic influences. Their greatest value is therefore mechanistic refinement before progression to animal or clinical studies.

### 6.4. Animal Models of Oral Mucosal Injury

Animal models remain important because they allow oral mucosal repair to be studied within an integrated biological system. Rodent oral wounds can be induced by excision, punch biopsy, incision, chemical injury, thermal injury, laser injury, or standardized ulceration at sites such as buccal mucosa, tongue, gingiva, or palate [5,10,11,35]. These models enable time-defined assessment of wound area, re-epithelialization, inflammatory infiltration, angiogenesis, collagen deposition, matrix remodeling, antioxidant responses, and local oxidative damage.

Impaired-healing models are especially relevant for redox-guided PBM research. Diabetes models, for example, reproduce several mechanisms that may delay repair, including mitochondrial dysfunction, endothelial impairment, reduced NO bioavailability, excessive ROS generation, inflammatory persistence, and delayed epithelial recovery [4,55,100,101,104,105]. A recent in vivo diabetic rat study of oral mucosal ulcer healing reported that PBM accelerated wound repair and favorably modified systemic oxidative-stress indices, illustrating how disease-context models can link macroscopic repair with redox outcomes [105].

Animal studies should report strain, sex, age, metabolic status, anesthesia, analgesia, housing, wound site, injury method, sample timing, randomization, blinding, sample-size justification, inclusion and exclusion criteria, and missing-data handling according to Animal Research: Reporting of In Vivo Experiments (ARRIVE) 2.0 principles [106]. When animal intervention studies are critically appraised, risk-of-bias domains such as allocation, blinding, attrition, selective reporting, and other design-related biases should also be considered [107].

### 6.5. Protocol Reporting and Dose Optimization

PBM protocols should be reported with enough detail to allow reconstruction of the optical exposure received by the tissue or culture. At minimum, studies should provide wavelength, light source, output power, irradiance, fluence, beam area at the target surface, exposure time, energy per point, number and location of irradiation points, contact or non-contact delivery, source-to-tissue distance, pulse settings, treatment frequency, number of sessions, cumulative exposure, and timing relative to injury [32,33,86]. These parameters are essential for reproducibility, dose–response interpretation, and comparison between studies. Jenkins and Carroll emphasize comparable core reporting items, including wavelength, output power, irradiation time, beam area, pulse parameters, anatomical location, number of treatments, and treatment interval. Table 4 translates these requirements into a practical PBM-redox reporting checklist.

Because PBM responses are often biphasic, dose reporting is central to interpretation. Subtherapeutic exposure may have little biological effect, an appropriate exposure range may promote adaptive ROS/NO signaling and repair, and excessive exposure may become inhibitory or pro-oxidant [83,84,85,86]. For this reason, dose optimization should be evaluated with biological outcomes rather than nominal fluence alone.

### 6.6. Clinical Study Designs

Clinical studies are needed to determine whether experimental PBM findings can be translated into oral wound care. Relevant settings include traumatic ulcers, surgical wounds, extraction sockets, periodontal and peri-implant soft-tissue wounds, oral mucositis, diabetic oral lesions, and chronic ulcerative conditions. In these settings, PBM outcomes should include not only closure or pain relief, but also inflammation, mucosal integrity, recurrence, functional recovery, and, where feasible, redox biomarkers.

Clinical biomarker studies require additional control of biological and preanalytical confounders. Saliva is non-invasive and directly relevant to the oral cavity, but its use in PBM trials should follow the standardized collection and processing principles summarized in Section 8.3. Tissue sampling provides more local information but is invasive, whereas blood reflects systemic redox status and should not be assumed to represent the oral wound microenvironment directly. Accordingly, clinical PBM studies should link biomarker changes with standardized clinical outcomes, wound photographs or measurements, pain scores where relevant, and tissue-level or surrogate repair-quality indicators.

### 6.7. Time Points and Outcome Integration

Sampling time should reflect the biology of repair. Early time points are most informative for hemostasis, inflammatory oxidant activity, MPO-related responses, cytokine release, neutrophil infiltration, TOS, and AOPP. Intermediate time points are better suited for epithelial migration, fibroblast activity, angiogenesis, TAC, antioxidant enzymes, Nrf2/HO-1, VEGF, and collagen deposition. Later time points should assess remodeling, MMP/TIMP balance, collagen organization, residual oxidative damage, epithelial maturation, and functional recovery.

Longitudinal designs are preferable because single measurements may miss the transition from adaptive redox signaling to persistent oxidative injury. For example, early increases in TOS, MPO-related activity, or AOPP may reflect inflammatory defense, whereas persistent MDA, protein carbonyls, 4-HNE, or 8-OHdG during later phases more strongly suggests unresolved tissue injury [18,19,20,23,51,52]. Histological and functional endpoints should be pre-specified to contextualize redox-marker trajectories and avoid overinterpreting isolated biochemical changes.

### 6.8. Common Limitations and Sources of Bias

Several factors reduce comparability across PBM and oxidative stress studies, including heterogeneous wound models, anatomical sites, irradiation parameters, treatment schedules, timing of irradiation, biomarker assays, control groups, randomization procedures, blinding, sample-size justification, and outcome definitions [32,106,107]. Many studies also assess only one or two oxidative stress markers, which is insufficient to characterize the redox environment or distinguish adaptive signaling from oxidative injury.

In many PBM studies, the main limitation is not the absence of positive findings, but the incomplete biological interpretation of those findings. Faster wound closure is often reported without enough information on epithelial maturation, inflammatory resolution, angiogenesis, collagen organization, oxidative damage, antioxidant recovery, or PBM dose distribution. This makes it difficult to distinguish accelerated closure from complete biological repair.

A redox-guided study design should combine adequate controls, transparent PBM dosimetry, appropriate sampling time points, balanced oxidative stress panels, tissue-level repair outcomes, and bias-reduction methods such as randomization, allocation concealment where applicable, blinded assessment, and predefined analysis plans. This approach would improve reproducibility and allow PBM effects to be interpreted as biochemical modulation of repair rather than as nonspecific acceleration of wound closure.

Representative evidence linking PBM parameters, oxidative stress biomarkers, and wound-repair outcomes is summarized in Table 5. The table is intended as a critical evidence map to illustrate how different experimental and clinical models contribute to the proposed framework, rather than as an exhaustive systematic synthesis or efficacy ranking.

## 7. Photobiomodulation Parameters and Biological Outcomes

### 7.1. Importance of Parameter-Dependent Interpretation

PBM outcomes depend on the interaction between light-delivery parameters and the biological condition of the target tissue. Wavelength, fluence, irradiance, exposure time, pulse mode, beam profile, spot size, energy per point, treatment interval, and cumulative exposure all shape the photochemical stimulus. However, the same nominal dose may produce different biological effects in healthy, diabetic, inflamed, infected, hypoxic, or therapy-injured oral mucosa [32,33,83,84,85,86,87,108]. PBM should therefore be interpreted as a parameter- and context-dependent intervention rather than as a uniform pro-healing stimulus.

### 7.2. Wavelength and Tissue Interaction

Wavelength determines how light is absorbed, scattered, and transmitted through oral soft tissues, and it influences interactions with mitochondrial and non-mitochondrial photoacceptors [24,25,27,28,33,108]. In oral mucosal applications, wavelength selection should therefore be considered together with mucosal thickness, pigmentation, hydration, vascularity, inflammation, anatomical site, and target depth [32,33,108,109]. These tissue-related factors help explain why similar wavelengths may produce different biological responses across experimental models and clinical wound phenotypes.

### 7.3. Fluence, Irradiance, and Exposure Time

Fluence, irradiance, and exposure time are closely related, but they are not interchangeable. The same fluence delivered at high irradiance over a short exposure period may not produce the same biological response as the same fluence delivered at lower irradiance over a longer period [33,83,84,86]. This distinction is especially important in redox biology, where the rate and duration of light exposure may influence mitochondrial activity, transient ROS/NO signaling, antioxidant adaptation, or, at excessive exposure levels, inhibitory and pro-oxidant responses. These parameters should therefore be interpreted together rather than reduced to nominal fluence alone, and their reporting should follow the checklist summarized in Table 4.

### 7.4. Treatment Timing and Number of Sessions

Treatment timing should follow the biology of repair. PBM applied during the early phase may influence inflammatory-cell activity, oxidative burst, antimicrobial defense, and the transition toward inflammatory resolution. During the proliferative phase, PBM may affect epithelial migration, fibroblast activity, angiogenesis, extracellular matrix deposition, and antioxidant adaptation. Later in repair, PBM may influence collagen maturation, matrix remodeling, and functional restoration [10,35,36,37,53]. Repeated sessions may be necessary to maintain a biologically relevant stimulus, but cumulative exposure should remain within the therapeutic window [83,84].

### 7.5. Wound Closure, Re-Epithelialization, and Repair Quality

Reduction in wound area is an important outcome, but it is not sufficient to define complete biological repair. Redox-guided PBM studies should combine macroscopic closure with indicators of repair quality, including epithelial continuity and thickness, inflammatory-cell infiltration, vascular density, collagen organization, matrix remodeling, persistent oxidative damage, and antioxidant recovery [5]. This broader approach helps distinguish accelerated surface closure from true restoration of mucosal structure and function.

### 7.6. Inflammation, Angiogenesis, and Matrix Remodeling

PBM may influence cytokine production, macrophage and neutrophil activity, nitric oxide availability, HIF-1α/VEGF signaling, endothelial function, collagen deposition, and MMP/TIMP balance [28,29,37,38,39,70]. A favorable PBM response should not be interpreted as complete suppression of inflammation or ROS production. Instead, it should reflect a timely transition from early host-defense signaling toward inflammatory resolution, angiogenesis, extracellular matrix organization, and barrier restoration.

### 7.7. Redox Outcomes and Biological Meaning

A favorable PBM-related redox response may include reduced persistent oxidant burden, lower lipid, protein, or DNA damage, improved antioxidant capacity, restored thiol balance, and activation of cytoprotective pathways, while preserving transient ROS signaling needed for repair [24,25,26,29]. Interpretation should remain phase-specific. Early ROS generation may represent adaptive signaling and antimicrobial defense, whereas persistent oxidative damage during proliferation or remodeling is more consistent with impaired resolution and delayed repair.

### 7.8. Toward Biologically Coherent PBM Protocols

Biologically coherent PBM protocols should be selected according to wound type, disease context, tissue depth, baseline redox state, healing phase, and intended molecular outcome. Complete dosimetry and integrated clinical, histological, biochemical, and molecular endpoints are needed to compare protocols and avoid interpreting PBM as a nonspecific accelerator of wound closure [32,33,108]. In this framework, the most informative PBM studies are those that link light-delivery parameters with redox trajectories, repair quality, and biological context.

## 8. Methodological Challenges in Redox Biomarker Assessment

### 8.1. Importance of Methodological Standardization

Redox biomarker values are highly method-dependent. The measured result can vary with sample matrix, collection procedure, processing delay, storage, assay platform, calibration, normalization, and reporting units [23,99]. This issue is especially important for oral and salivary samples, where biological variability and preanalytical conditions can strongly influence oxidative stress markers [16,23,40].

In PBM studies, methodological transparency is essential because biomarker responses depend not only on disease state and healing phase, but also on irradiation variables and baseline tissue status [32]. Without standardized sampling and complete PBM reporting, it is difficult to determine whether differences between studies reflect true biological effects, preanalytical variability, assay limitations, or incomplete protocol description.

### 8.2. Choice of Biological Sample

Sample choice should match the research question. Tissue provides the most direct information on local oxidative damage, antioxidant defense, inflammation, angiogenesis, matrix remodeling, and pathway activation, but it is invasive and difficult to collect repeatedly in clinical studies. Saliva is non-invasive and directly relevant to the oral cavity, reflecting epithelial turnover, microbial activity, inflammation, antioxidant defense, and local redox status [16,23,40,98]. However, saliva is a mixed biological fluid and is sensitive to both local and systemic influences.

Blood samples may capture systemic oxidative stress in diabetes, cancer therapy, chronic inflammation, or metabolic disease, but they should not be assumed to represent the oral wound microenvironment directly. Tissue, saliva, and blood should be interpreted as complementary rather than equivalent matrices. The strongest designs are those in which the selected sample type is clearly linked to the biological hypothesis, the wound model, and the intended translational application.

### 8.3. Preanalytical Sources of Variability

Preanalytical variability is a major limitation in redox biomarker studies. Salivary studies should standardize unstimulated versus stimulated collection, whole versus gland-specific saliva, time of day, fasting status, oral hygiene conditions, posture, hydration, periodontal status, recent dental procedures, smoking, medication use, and blood contamination [16,23,40]. These variables are particularly important when saliva is used to monitor PBM effects because small treatment-related changes may be obscured by collection-related variability.

Tissue studies should report anatomical site, wound region, sampling depth, time after injury, handling time, washing procedure, homogenization method, buffer composition, temperature control, and normalization strategy. Blood studies should control serum versus plasma choice, anticoagulant, centrifugation protocol, hemolysis, storage temperature, storage duration, and freeze–thaw cycles. These details are not minor technical issues; they directly affect the reliability and comparability of oxidative stress measurements.

### 8.4. Analytical Method Selection

Analytical methods should be chosen according to the specific research question, with attention to specificity, sensitivity, reproducibility, sample compatibility, and biological relevance. TOS and TAC are useful broad measures but cannot identify individual oxidants, antioxidant systems, or molecular injury targets [14,15]. MDA assays, especially TBARS-based methods, require cautious interpretation because other reactive compounds can contribute to the signal [18,102]. Protein carbonyls, AOPP, and 8-OHdG represent different forms of molecular damage and should not be treated as interchangeable endpoints [20,52,54].

Assay-level choices should be explicitly justified. MDA can be measured by TBARS, high-performance liquid chromatography (HPLC), or liquid chromatography–mass spectrometry (LC-MS)-based methods; TBARS is accessible but less specific, whereas chromatographic approaches improve specificity when lipid peroxidation is a central endpoint. 4-HNE can be assessed by enzyme-linked immunosorbent assay (ELISA), immunohistochemistry, or chromatographic methods, but tissue localization and adduct specificity differ between platforms. AOPP is commonly measured spectrophotometrically and is informative for chlorinated oxidant and MPO-related activity, but it is sensitive to sample handling, turbidity, hemolysis, and protein concentration. Protein carbonyls may be quantified spectrophotometrically, fluorometrically, or immunochemically and should be normalized consistently to protein content.

For oxidative DNA damage, 8-OHdG measured by ELISA is practical for larger studies but may be less specific than high-performance liquid chromatography with electrochemical detection (HPLC-ECD) or liquid chromatography–tandem mass spectrometry (LC-MS/MS). Therefore, safety-oriented or mechanistic studies should consider more specific approaches when feasible. TOS, TAC, and OSI require careful attention to assay platform, calibration, units, and calculation formulas, because OSI values are not directly comparable if component units differ. SOD, CAT, and GPx activities should be reported with tissue homogenate normalization, protein assay method, enzyme stability conditions, and temperature control. Thiol/disulfide measurements are particularly vulnerable to ex vivo oxidation; rapid processing, controlled storage, and clear reporting of native thiol, total thiol, disulfide, and ratio formulas are essential [14,15,16,17,23,99,102].

Studies should report assay type, manufacturer, calibration procedure, analytical range, intra- and inter-assay variability, units, sample dilution, sample handling, storage conditions, and exclusion criteria for poor-quality samples. When calculated indices such as OSI or thiol/disulfide parameters are used, formulas, units, and conversion steps should be explicitly described [14,15,17]. This level of detail is essential for reproducibility and meaningful comparison across PBM protocols.

### 8.5. Normalization and Data Expression

Normalization is essential for reliable interpretation. Tissue biomarkers are commonly expressed relative to total protein, tissue weight, or sample volume, while enzyme activities may be expressed per milligram of protein, gram of tissue, or fluid volume. In saliva, concentration alone may be misleading when flow rate, dilution, or protein content differs between groups. Secretion rate or selected normalization approaches may therefore be considered, although total protein itself can change with inflammation, epithelial shedding, microbial load, or blood contamination.

Data expression should be consistent within a study and transparent enough to allow comparison with other reports. For derived measures such as OSI, reduced and oxidized thiol fractions, or thiol/disulfide ratios, authors should specify the mathematical derivation and ensure that component variables are measured in compatible units [14,15,17]. Without clear normalization and reporting, apparent differences in redox status may reflect sample dilution, tissue composition, or calculation artifacts rather than biological change.

### 8.6. Timing of Sampling

From an analytical perspective, sampling time should be matched to the dominant biological events in the wound. Early sampling is most informative for inflammatory oxidant activity, MPO-related responses, cytokine release, neutrophil infiltration, TOS, and AOPP. Mid-phase sampling is better suited for epithelial migration, fibroblast activity, angiogenesis, TAC, antioxidant enzyme responses, Nrf2/HO-1, VEGF, and collagen deposition. Late sampling should focus on remodeling, MMP/TIMP balance, collagen architecture, residual oxidative damage, epithelial maturation, and functional recovery.

Longitudinal designs are preferable to single-endpoint measurements because they can distinguish transient adaptive ROS signaling from persistent oxidative injury. A single elevated oxidant marker may have different meanings depending on whether it occurs during early inflammation or delayed remodeling. Redox biomarker trajectories should therefore be interpreted together with wound phase, PBM timing, disease context, and histological or clinical outcomes.

### 8.7. Integration with Histological and Clinical Outcomes

Outcome integration prevents biomarker overinterpretation. Reduced MDA, TOS, AOPP, protein carbonyls, or 8-OHdG is more convincing when accompanied by epithelial maturation, inflammatory resolution, improved vascular response, organized collagen deposition, and restored barrier function. Conversely, improved closure with persistent oxidative damage or poor matrix organization should be interpreted cautiously.

Useful tissue-level endpoints include epithelial continuity, epithelial thickness, inflammatory-cell infiltration, fibroblast activity, vascular density, collagen organization, matrix remodeling, apoptosis/proliferation markers, Nrf2/HO-1, NF-κB, VEGF, and MMP/TIMP balance [37,38,39,64,65,69,70,71]. Clinical endpoints may include standardized wound area, pain, erythema, edema, mucosal integrity, time to closure, and recurrence where relevant. In PBM studies, biochemical, histological, and clinical outcomes should be interpreted as a coordinated pattern rather than as independent proof of efficacy.

### 8.8. Statistical and Reporting Considerations

Redox biomarker studies often include multiple markers, time points, and groups, increasing the risk of selective interpretation and false-positive findings. Statistical analysis should reflect data distribution, sample size, experimental design, repeated measurements where applicable, and the prespecified biological hypothesis. Isolated significant results should not be overstated when the broader biomarker pattern, histology, or clinical outcome is inconsistent.

Experimental animal studies should report allocation, randomization, blinding, sample-size justification, exclusion criteria, missing-data handling, animal characteristics, housing, injury method, and timing of intervention and sampling according to ARRIVE 2.0 principles [106]. Risk-of-bias domains such as allocation, blinding, attrition, selective reporting, and other design-related biases should also be considered when animal intervention studies are interpreted [107]. Clinical studies should report eligibility criteria, wound type, systemic conditions, medication use, oral hygiene, smoking, PBM protocol, sham procedure where applicable, predefined outcomes, and CONSORT- or STROBE-relevant details according to study design [110,111].

### 8.9. Toward Reproducible Redox-Guided PBM Research

Future PBM studies should use redox-guided designs based on standardized sampling, justified biomarker selection, complete irradiation reporting, and biologically relevant models [32,99]. A practical strategy is to combine one global redox marker, one molecular damage marker, one antioxidant defense marker, one pathway-related marker, and one repair-quality outcome, then interpret these variables according to wound phase and disease context.

This framework would improve comparability across studies and reduce overinterpretation of isolated redox changes. Declines in MDA or TOS, for example, are most meaningful when accompanied by epithelial maturation, reduced persistent inflammation, restored antioxidant reserve, and organized matrix remodeling; similarly, activation of Nrf2/HO-1 or VEGF should be linked to antioxidant adaptation, angiogenesis, and repair quality. Such an approach should be viewed as an interpretative and study-design framework rather than as a validated clinical decision algorithm.

## 9. Translational Perspectives and Future Directions

### 9.1. From Experimental Findings to Translational Relevance

The translational value of redox biomarkers depends on whether they connect biochemical change with clinically meaningful repair. Faster wound closure after PBM is encouraging, but it does not by itself prove restoration of mucosal biology. A more informative approach asks whether PBM reduces persistent oxidative injury, supports antioxidant recovery, improves inflammatory resolution, promotes angiogenesis, restores epithelial barrier function, and improves patient-relevant outcomes.

A translationally useful PBM study should ask not only whether the wound closes faster, but also whether the tissue recovers in a coordinated way. This requires linking PBM parameters with biomarker patterns, histological repair, pain or functional outcomes, recurrence risk, and safety. Such integration is particularly important in patients with diabetes, cancer therapy-related mucositis, chronic inflammation, or infection, where the baseline redox environment is already disturbed.

At the present stage, redox-guided PBM should be considered a research and interpretation framework rather than a clinical decision-making tool. Biomarker patterns may help generate hypotheses, compare protocols, and identify repair phenotypes, but they are not yet validated for selecting PBM dose, predicting individual response, or guiding routine clinical decisions.

### 9.2. Redox-Guided Optimization of PBM Protocols

A major future direction is redox-guided PBM optimization. Heterogeneous irradiation parameters limit comparisons between studies, and the biphasic PBM response means that wound closure alone is insufficient for dose selection [32,83,84,86]. Future protocols should test how wavelength, irradiance, fluence, exposure time, treatment frequency, and cumulative dose affect TOS, TAC, OSI, molecular damage markers, antioxidant defense, pathway activation, and repair quality across defined healing phases.

This approach would allow PBM protocols to be compared according to biological response patterns rather than nominal dose or clinical improvement alone. A protocol that reduces persistent oxidative damage, restores antioxidant reserve, supports angiogenesis, and improves epithelial and matrix organization is more informative than a protocol that reports closure alone.

### 9.3. Stratification According to Wound Phenotype and Baseline Redox Status

Clinical oral wounds differ substantially in baseline biology. An acute traumatic ulcer in an otherwise healthy individual does not have the same redox environment as a diabetic wound, chemotherapy-induced mucositis, radiotherapy-associated lesion, periodontal wound, infected lesion, or chronic ulcer [3,4,43,44,45,55]. Baseline biomarker assessment may help identify high oxidant burden, antioxidant depletion, persistent protein oxidation, lipid peroxidation, DNA damage, or unresolved inflammatory activity.

Future PBM studies should consider stratifying participants or experimental models according to wound phenotype and baseline redox status. Such stratification may help explain why similar irradiation protocols produce different outcomes across disease contexts and may support more individualized PBM dose selection.

### 9.4. Clinical Contexts with High Translational Potential

High-priority clinical contexts include chemotherapy- and radiotherapy-induced oral mucositis, diabetes-associated oral wounds, surgical wounds, extraction sockets, periodontal wounds, peri-implant soft-tissue lesions, traumatic ulcers, recurrent aphthous stomatitis and other recurrent ulcerative conditions, infected lesions, and chronic inflammatory mucosal disease [3,4,43,44,45,55,112]. These settings are suitable for testing whether PBM improves not only wound closure or pain, but also redox balance, epithelial restoration, vascular response, matrix remodeling, and barrier function.

Cancer therapy-induced oral mucositis is particularly relevant because oxidative stress, epithelial injury, inflammation, pain, and barrier disruption are central to its pathobiology [3,43,44,45]. Diabetes-associated wounds are also important because mitochondrial dysfunction, endothelial impairment, reduced nitric oxide bioavailability, and persistent oxidative stress may alter PBM responsiveness [4,55,104].

However, the strength of evidence differs substantially between these indications. PBM protocols validated for oral mucositis should not be directly extrapolated to diabetic, infected, traumatic, periodontal, or peri-implant wounds without considering tissue depth, wound biology, microbial burden, vascular status, baseline redox state, and treatment goals.

### 9.5. Integrating Salivary Biomarkers into Clinical PBM Studies

Saliva is a promising non-invasive matrix because it is easy to collect and directly related to the oral cavity [16,23,40,112,113]. Potential markers include TOS, TAC, OSI, MDA, 4-HNE, AOPP, protein carbonyls, 8-OHdG, SOD, GPx, thiol/disulfide balance, and inflammatory mediators. Their use in PBM studies should follow the preanalytical standardization principles outlined in Section 8.3, without repeating caveat lists or relying on unstandardized sampling.

For translational PBM studies, saliva should not be treated as a simple substitute for tissue. It is better used as a complementary matrix that may reflect oral redox status, inflammatory activity, and treatment response when collection and interpretation are standardized. Whenever possible, salivary biomarkers should be linked to clinical scoring, wound photographs, histology, pain, function, and treatment timing.

### 9.6. Toward Integrated Outcome Frameworks

Current PBM literature often separates clinical, histological, biochemical, and molecular outcomes. Integrated frameworks should assess wound area, closure time, pain, erythema, edema, function, epithelial continuity, inflammatory infiltration, vascularity, collagen deposition, matrix remodeling, redox biomarkers, cytokines, angiogenic factors, and signaling pathways [5,37,38,39,40,64,65,69,70,71]. Such integration helps distinguish superficial closure from genuine repair quality.

This integrated approach is particularly important in redox-guided PBM research because the desired outcome is coordinated biological repair: controlled inflammatory resolution, reduced persistent molecular damage, restored antioxidant buffering, appropriate angiogenesis, organized extracellular matrix remodeling, and functional mucosal recovery.

As summarized in Figure 4, redox-guided PBM studies should integrate wound phenotype, healing phase, PBM parameters, sample matrix, biomarker category, redox-sensitive pathways, and repair-quality outcomes rather than assessing clinical closure, oxidative-stress markers, or molecular pathways in isolation.

### 9.7. Requirements for Future Experimental and Clinical Studies

Future studies should improve reproducibility through complete PBM reporting, standardized biomarker methodology, appropriate controls, randomization, blinding, sample-size justification, sham-controlled clinical designs, predefined outcomes, and biologically meaningful time points [32,106,107,110,111]. PBM protocols should follow the reporting checklist summarized in Table 4, while biomarker methodology should be described with similar precision.

Authors should report sample type, collection conditions, processing time, storage, normalization, assay platform, calibration, intra- and inter-assay variability, units, and formulas for calculated indices such as OSI or thiol/disulfide parameters [14,15,16,17,23,99,102]. Without this level of detail, inconsistent findings cannot be confidently attributed to biological variability or PBM protocol differences.

Recommended context-specific design elements for future redox-guided PBM studies in oral mucosal wound healing are summarized in Table 6.

### 9.8. Future Research and Translational Priorities

Future research should move from isolated biomarker or wound-closure endpoints toward validation of compact, phase-specific PBM-redox panels. Priority areas include defining dose–phase interactions; validating tissue, saliva, serum, and plasma biomarker panels; standardizing salivary collection and analytical methodology; and integrating Nrf2/HO-1, NF-κB, VEGF, NOX, MMP/TIMP, nitrite/nitrate, and mitochondrial endpoints with epithelial maturation, inflammatory resolution, angiogenesis, matrix organization, and barrier recovery.

Translationally, oxidative stress biomarkers are most useful when they help determine whether a PBM protocol is biologically rational, reproducible, and clinically meaningful. They should therefore be embedded in study designs that combine complete PBM dosimetry, wound phenotype, sample matrix, healing phase, pathway activation, and repair-quality outcomes, rather than being used as isolated laboratory readouts.

## 10. Limitations of This Narrative Review

This narrative review does not provide systematic evidence grading or quantitative synthesis and therefore cannot determine the comparative efficacy of specific PBM protocols across oral wound indications. The included literature is heterogeneous with respect to wound model, disease context, PBM parameters, sample matrix, biomarker panel, timing of assessment, and outcome definition. Several mechanisms discussed in this review, particularly cysteine-specific redox switching, mitophagy, macrophage immunometabolism, inflammasome persistence, ferroptosis, extracellular-vesicle signaling, and omics-defined redox states, remain extrapolated or hypothesis-generating unless they are directly demonstrated in oral mucosal PBM models.

Accordingly, the strongest conclusions of this review concern general redox principles, biomarker limitations, methodological requirements, and the need for complete PBM reporting. Emerging molecular mechanisms should be viewed as priorities for future validation rather than as established clinical mechanisms. The proposed framework is intended to improve study design and interpretation, but it does not yet represent a validated diagnostic, prognostic, or therapeutic algorithm.

## 11. Conclusions

This review proposes a redox-guided interpretative framework for oral mucosal PBM research that links PBM dosimetry, healing phase, sample matrix, biomarker category, redox-sensitive pathways, and repair-quality outcomes.

By shifting the emphasis from isolated wound closure or single oxidative-stress markers to integrated repair quality, the framework may help standardize biomarker selection, improve PBM protocol reporting, distinguish adaptive ROS/NO signaling from persistent oxi-inflammatory injury, and separate established mechanisms from plausible but still unvalidated hypotheses. Future studies should test whether this approach improves assessment of epithelial maturation, inflammatory resolution, vascular support, extracellular matrix organization, barrier recovery, and control of persistent oxidative damage.

## Figures and Tables

**Table 1:** Conceptual contribution of the present review compared with previous reviews on oral wound healing, oxidative stress, and photobiomodulation.

Previous Focus	Limitation	Added Value of This Review
Oral wound-healing biology	Limited integration of redox biomarkers and PBM parameters	Links repair phases with redox biomarker interpretation
Oral wound models and outcome measures	Closure is often emphasized more than repair quality	Connects epithelial closure with histological repair, matrix remodeling, inflammation resolution, and barrier restoration
PBM efficacy studies	Often focused on closure, pain, or clinical improvement	Adds PBM dosimetry, biological timing, and the redox therapeutic window
Oxidative stress biomarkers	Often interpreted marker by marker	Integrates TOS/TAC/OSI, oxidative damage markers, antioxidant defense, and redox-sensitive pathways
Salivary biomarkers	Preanalytical and biological variability	Proposes a sampling and interpretation framework based on sample type, disease context, and healing phase

The table summarizes the main conceptual gaps addressed by this review. It is intended to clarify the added value of the proposed redox-guided interpretative framework and does not represent a systematic ranking of previous reviews or a quantitative evidence assessment. PBM, photobiomodulation; TOS, total oxidant status; TAC, total antioxidant capacity; OSI, oxidative stress index.

**Table 2:** Critical interpretation of oxidative stress biomarkers in oral wound-healing and photobiomodulation studies: main value, limitations, and recommended use in PBM-oriented research.

Biomarker Group	Main Value	Main Limitation	Best Use in PBM Studies
TOS/TAC/OSI	Global direction of oxidant–antioxidant balance	Nonspecific; does not identify oxidant source or molecular target	Screening overall redox shift across groups, time points, or PBM protocols
MDA	Common lipid peroxidation marker	Thiobarbituric acid- reactive substances (TBARS)-based methods have specificity limitations	Use with 4-HNE, protein oxidation, DNA damage, or histological outcomes
4-HNE	Mechanistic lipid peroxidation product and bioactive aldehyde	Less commonly measured; method- dependent	Stronger interpretation of lipid-derived oxidative injury and signaling
AOPP	Protein oxidation linked to chlorinated oxidants and MPO-related inflammation	Influenced by inflammatory activity and sample handling	Early inflammatory oxidative activity and neutrophil/MPO-associated stress
Protein carbonyls	Broad measure of oxidative protein damage	Limited pathway specificity	Persistent oxidative protein damage during delayed healing
8-OHdG	Oxidative DNA damage	Not a general wound- closure marker	Mucositis, radiation injury, chronic inflammation, safety, and repair-quality assessment
SOD/CAT/GPx	Enzymatic antioxidant defense	Adaptive activation and exhaustion can be difficult to distinguish	Interpret with TOS/TAC/OSI and molecular damage markers
GSH/ thiol-disulfide balance	Redox buffering and thiol homeostasis	Preanalytical sensitivity; normalization issues	Recovery of antioxidant reserve and redox buffering after PBM
Nrf2/HO-1, NF-κB, VEGF, NOX, MMP/TIMP	Pathway-level mechanism	Requires tissue/cellular context; marker meaning is pathway- and phase-dependent	Linking redox changes with inflammation, angiogenesis, cytoprotection, and remodeling

Biomarker groups are presented according to their main interpretative value in oral wound-healing and PBM studies. None of these markers should be interpreted in isolation; results should be evaluated together with sample type, healing phase, disease context, PBM parameters, and tissue-level repair outcomes. AOPP, advanced oxidation protein products; CAT, catalase; GPx, glutathione peroxidase; GSH, glutathione; HO-1, heme oxygenase-1; MDA, malondialdehyde; MMP, matrix metalloproteinase; MPO, myeloperoxidase; NF-κB, nuclear factor kappa B; NOX, NADPH oxidase; Nrf2, nuclear factor erythroid 2-related factor 2; OSI, oxidative stress index; PBM, photobiomodulation; TAC, total antioxidant capacity; TBARS, thiobarbituric acid-reactive substances; TIMP, tissue inhibitor of metalloproteinases; TOS, total oxidant status; VEGF, vascular endothelial growth factor; 4-HNE, 4-hydroxynonenal; 8-OHdG, 8-hydroxy-2′-deoxyguanosine.

**Table 3:** Recommended minimal and extended biomarker panels according to study aim in photobiomodulation-oriented oral mucosal wound-healing research.

Study Aim	Minimal Panel	Extended Panel	Preferred Sample/Outcome Link
Basic oral wound model	TOS/TAC/OSI + MDA or 4-HNE + SOD/CAT/GPx + histology	AOPP/protein carbonyls, Nrf2/HO-1, NF-κB, VEGF, MMP/TIMP	Tissue; link redox changes with re-epithelialization, inflammation, angiogenesis, and collagen organization
Salivary clinical monitoring	TOS/TAC/OSI + AOPP or MDA + thiol/disulfide balance + standardized clinical wound score	8-OHdG, protein carbonyls, inflammatory mediators, selected antioxidant enzymes	Saliva; control flow rate, circadian timing, oral hygiene, periodontal status, blood contamination, smoking, and medication use
Mucositis or radiotherapy injury	MDA/4-HNE + protein carbonyls or AOPP + 8-OHdG + pain/mucositis severity score	NF-κB, Nrf2/HO-1, apoptosis/proliferation markers, VEGF, cytokines	Tissue and/or saliva; link biomarkers with epithelial injury, ulcer severity, pain, and barrier recovery
Diabetes-associated oral wounds	TOS/TAC/OSI + MDA/4-HNE + SOD or GPx + wound area/histology	NOX, nitrite/nitrate, VEGF, Nrf2/HO-1, MMP/TIMP, thiol/disulfide balance	Tissue, saliva, and/or blood; interpret in relation to glycemic/metabolic status and vascular repair
PBM dose optimization	One global redox marker + one damage marker + one antioxidant defense marker + wound repair endpoint	Nrf2/HO-1, NF-κB, HIF-1α/VEGF, NOX, MMP/TIMP, mitochondrial or NO-related markers	Matrix selected by model; evaluate together with wavelength, irradiance, fluence, exposure time, treatment schedule, and cumulative dose

Minimal panels are intended to provide a practical core set of complementary markers, whereas extended panels may be added when the study design, sample availability, and mechanistic question allow broader pathway-level interpretation. Biomarker selection should remain hypothesis-driven and should be interpreted together with PBM parameters, wound phase, sample type, disease context, and tissue-level or clinical repair outcomes. AOPP, advanced oxidation protein products; CAT, catalase; GPx, glutathione peroxidase; HIF-1α, hypoxia-inducible factor-1 alpha; HO-1, heme oxygenase-1; MDA, malondialdehyde; MMP, matrix metalloproteinase; NF-κB, nuclear factor kappa B; NOX, NADPH oxidase; Nrf2, nuclear factor erythroid 2-related factor 2; OSI, oxidative stress index; PBM, photobiomodulation; TAC, total antioxidant capacity; TIMP, tissue inhibitor of metalloproteinases; TOS, total oxidant status; VEGF, vascular endothelial growth factor; 4-HNE, 4-hydroxynonenal; 8-OHdG, 8-hydroxy-2′-deoxyguanosine.

**Table 4:** Practical photobiomodulation-redox reporting checklist for oral mucosal wound-healing studies.

Item Group	Details to Include	Interpretive Value
Light source and beam	Device or light source; wavelength; output power; beam area measured at the target surface; spot size; continuous or pulsed mode and pulse settings.	Clarifies the physical stimulus delivered to tissue and permits comparison of photon delivery between studies.
Dose delivery	Irradiance; fluence; energy per point; exposure duration; number and location of points; contact or non-contact application; source-to-tissue distance.	Distinguishes total exposure from rate of energy delivery and helps explain biphasic, absent, inhibitory, or pro-oxidant responses.
Treatment schedule	Time of first irradiation relative to injury; session frequency; total number of sessions; interval between sessions; cumulative exposure.	Relates PBM exposure to inflammatory, proliferative, and remodeling phases rather than treating treatment timing as interchangeable.
Tissue and model context	Anatomical wound site; target depth or tissue thickness when available; disease or injury phenotype; baseline metabolic/redox status; sham or control condition.	Helps explain why similar optical parameters may produce different biological responses in healthy, diabetic, inflamed, or therapy-injured oral tissues.
Outcome integration	Wound area, re-epithelialization, histological repair quality, inflammation, angiogenesis, matrix remodeling, and predefined biomarker panel.	Reduces the risk of judging PBM efficacy from surface closure or a single oxidative-stress marker.

Note: This checklist is intended to improve reproducibility, dose–response interpretation, and biological comparison across PBM studies. It should not be interpreted as a universal therapeutic protocol. Reported parameters should be evaluated together with wound model, anatomical site, tissue depth, disease context, healing phase, sample type, and clinical, histological, biochemical, or molecular outcomes. PBM, photobiomodulation.

**Table 5:** Representative evidence linking photobiomodulation, oxidative stress biomarkers, and wound-repair outcomes.

Model/Condition	PBM Parameters/ Dose-Reporting Relevance	Sample/ Biomarkers	Main Redox and Repair Finding	Main Limitation	Framework Interpretation
Oral mucosal ulcers in healthy and diabetic rats [105]	630–660 nm; 7 mW; 0.196 cm^2^ probe; 6 J/cm^2^; continuous mode; every other day for 10 days	Plasma/systemic markers and wound assessment; TOS, TAC, OSI, SOD	Context- and time-dependent redox improvement accompanied by faster oral ulcer healing, especially in diabetes	Systemic indices; limited local tissue and pathway profiling	Direct oral relevance; local tissue and pathway validation still needed
Diabetic wounded fibroblast model [63]	Model-specific exposure reported in source	Diabetic wound fibroblasts; oxidative-stress and FOXO1- related markers	Reduced oxidative stress through FOXO1-related mechanisms, supporting fibroblast repair signaling	In vitro; not oral-specific; lacks tissue, saliva, and microbiome context	Mechanistic support; oral tissue validation needed
Diabetic neuropathic wound/ulcer models in Wistar rats [64,65]	Dose–response protocols, approximately 4–8 J/cm^2^	Animal tissue/systemic matrices; oxidative-stress and antioxidant indices	Dose-dependent redox modulation associated with improved diabetic wound healing	Cutaneous/ neuropathic model; oral transfer uncertain	Supports wound- model and dose–response concepts, but disease and tissue specificity remain important
Cancer therapy- induced oral mucositis PBM evidence [45]	Multiple wavelengths and doses across included studies	Clinical oral mucositis outcomes; redox biomarkers variably reported or often absent	Clinical benefit is clearer than the underlying redox mechanism, with reduced mucositis severity and/or pain	Limited integration of oxidative-stress, pathway, and complete dosimetry data	Clinical anchor; needs biomarker integration with complete PBM reporting
PBM effects on keratinocyte inflammatory response [82]	Study-specific exposure in an anti- inflammatory model	Keratinocyte/immune- cell model; Nrf2 and inflammatory markers	Cytoprotective signaling and reduced inflammatory response support epithelial stress modulation	Not a complete oral wound model	Supports the Nrf2/HO-1 cytoprotection versus persistent NF-κB inflammatory branch
PBM optimization for angiogenesis and mitochondrial function [108]	Radiometric and spectral parameters optimized in vitro	Angiogenesis/ mitochondrial model; mitochondrial and angiogenesis endpoints	Improved mitochondrial and angiogenic readouts support vascular repair mechanisms	In vitro; not oral-specific; limited oxidative- damage profiling	Links PBM parameters with mitochondrial and VEGF-related repair mechanisms
Dental fibroblast PBM parameter literature [89]	Variable dental PBM parameters across studies	Fibroblast studies; proliferation/ differentiation endpoints, with variable redox- marker inclusion	Responses depend on laser parameters, with improved fibroblast proliferation and/or differentiation reported in selected conditions	High heterogeneity; incomplete redox and repair-quality integration	Demonstrates the need to couple PBM dosimetry with redox and repair- quality outcomes

Note: This table is intended as a critical evidence map rather than an exhaustive systematic synthesis or efficacy ranking. PBM parameters are summarized to indicate dose-reporting relevance and should be interpreted together with the full protocols provided in the cited sources. Evidence from non-oral wound models, in vitro studies, and broader mechanistic systems is included as translational or mechanistic support and should not be interpreted as direct confirmation in oral mucosal PBM. Abbreviations: FOXO1, forkhead box protein O1; HO-1, heme oxygenase-1; NF-κB, nuclear factor kappa B; Nrf2, nuclear factor erythroid 2-related factor 2; OSI, oxidative stress index; PBM, photobiomodulation; SOD, superoxide dismutase; TAC, total antioxidant capacity; TOS, total oxidant status; VEGF, vascular endothelial growth factor.

**Table 6:** Recommended design elements for future redox-guided PBM studies in oral mucosal wound healing.

Study Context	Preferred Sample	Minimum Biomarker Panel	PBM Reporting Essentials	Preferred Outcome Measures	Main Interpretation Risk
Acute oral ulcer model	Tissue homogenate or local biopsy; saliva as adjunct in clinical studies	TOS/TAC/OSI + MDA or 4-HNE + SOD/CAT/GPx	Wavelength, output power, beam area, irradiance, fluence, exposure time, delivery mode, timing after injury	Wound area, re-epithelialization, inflammatory infiltrate, epithelial thickness, collagen organization	Mistaking faster surface closure for complete repair
Diabetic oral wound model	Tissue plus blood/serum; saliva when clinically feasible	TOS/TAC/OSI + MDA/4-HNE + SOD or GPx + thiol/disulfide balance	Dose per point, cumulative dose, number of sessions, timing relative to ulcer induction, metabolic status	Wound closure, histology, angiogenesis/ VEGF, collagen maturation, glycemic context	Attributing delayed healing only to local redox imbalance while ignoring systemic metabolic control
Oral mucositis model	Tissue and/or saliva; blood for systemic inflammatory context	MDA/4-HNE + protein carbonyls or AOPP + 8-OHdG + antioxidant defense marker	Full dosimetry, treatment schedule, anatomical site, mucositis grade, concomitant therapy	Mucositis severity, pain, ulcer duration, epithelial integrity, barrier recovery	Overgeneralizing protocols across chemotherapy and radiotherapy settings
Salivary biomarker clinical monitoring	Unstimulated saliva under standardized collection conditions	TOS/TAC/OSI + AOPP or MDA + thiol/disulfide balance	Sampling time, oral hygiene, fasting/hydration, periodontal status, blood contamination, PBM schedule	Clinical wound score, pain/ function, serial photographs, healing time	Treating saliva as a direct substitute for wound tissue
PBM dose- optimization study	Model-dependent matrix: tissue for mechanisms, saliva for translation, blood for systemic context	One global redox marker + one damage marker + one antioxidant marker + one pathway marker	Wavelength, irradiance, fluence, exposure time, beam area, energy per point, pulse mode, sessions, cumulative dose	Dose–response curve, histology, repair quality, pathway activation, adverse/pro-oxidant signals	Selecting dose based on closure alone rather than integrated redox and repair-quality endpoints

Note: This table summarizes study-design priorities for future experimental and clinical research. The proposed biomarker panels and outcome measures are intended to guide protocol development and interpretation, not to define validated diagnostic, prognostic, or therapeutic algorithms. Biomarker selection should be adapted to the wound phenotype, sample availability, PBM protocol, healing phase, and mechanistic hypothesis. AOPP, advanced oxidation protein products; CAT, catalase; GPx, glutathione peroxidase; MDA, malondialdehyde; OSI, oxidative stress index; PBM, photobiomodulation; SOD, superoxide dismutase; TAC, total antioxidant capacity; TOS, total oxidant status; VEGF, vascular endothelial growth factor; 4-HNE, 4-hydroxynonenal; 8-OHdG, 8-hydroxy-2′-deoxyguanosine.

## Data Availability

No original research data or new datasets were generated in this narrative review. The article is based on previously published literature, and data sharing is not applicable.

## Abbreviations

4-HNE, 4-hydroxynonenal; 8-OHdG, 8-hydroxy-2′-deoxyguanosine; ACSL4, acyl-CoA synthetase long-chain family member 4; AOPP, advanced oxidation protein products; ATP, adenosine triphosphate; CAT, catalase; CCO, cytochrome c oxidase; CD, cluster of differentiation; DNA, deoxyribonucleic acid; DRP1, dynamin-related protein 1; ECM, extracellular matrix; ERK, extracellular signal-regulated kinase; EV, extracellular vesicle; GPx, glutathione peroxidase; GPX4, glutathione peroxidase 4; GSH, glutathione; HIF-1α, hypoxia-inducible factor-1 alpha; HO-1, heme oxygenase-1; IL, interleukin; iNOS, inducible nitric oxide synthase; LC3, microtubule-associated protein 1 light chain 3; LLLT, low-level laser therapy; MAPK, mitogen-activated protein kinase; MDA, malondialdehyde; MFN, mitofusin; miRNA, microRNA; MMP, matrix metalloproteinase; MPO, myeloperoxidase; mtDNA, mitochondrial DNA; NADPH, nicotinamide adenine dinucleotide phosphate; NF-κB, nuclear factor kappa B; NLRP3, NOD-like receptor family pyrin domain-containing 3; NO, nitric oxide; NOX, NADPH oxidase; Nrf2, nuclear factor erythroid 2-related factor 2; OSI, oxidative stress index; PBM, photobiomodulation; PI3K/Akt, phosphoinositide 3-kinase/protein kinase B; PINK1, PTEN-induced kinase 1; RAS, recurrent aphthous stomatitis; ROS, reactive oxygen species; SLC7A11/xCT, solute carrier family 7 member 11/cystine-glutamate antiporter; SOD, superoxide dismutase; TAC, total antioxidant capacity; TAS, total antioxidant status; TGF-β, transforming growth factor beta; TIMP, tissue inhibitor of metalloproteinases; TOS, total oxidant status; VEGF, vascular endothelial growth factor.
